# Gene editing and cardiac disease modelling for the interpretation of genetic variants of uncertain significance in congenital heart disease

**DOI:** 10.1186/s13287-023-03592-1

**Published:** 2023-12-05

**Authors:** Vanessa S. Fear, Catherine A. Forbes, Nicole C. Shaw, Kathryn O. Farley, Jessica L. Mantegna, Jasmin P. Htun, Genevieve Syn, Helena Viola, Henrietta Cserne Szappanos, Livia Hool, Michelle Ward, Gareth Baynam, Timo Lassmann

**Affiliations:** 1 Translational Genetics, Precision Health, Telethon Kids Institute, Perth Children’s Hospital, Nedlands, WA 6009 Australia; 2 Computational Biology, Precision Health, Telethon Kids Institute, Perth Children’s Hospital, Nedlands, WA 6009 Australia; 3 Centre for Child Health Research, University of Western Australia, Crawley, Australia; 4 University of Western Australia, Crawley, Australia; 5 Victor Chang Cardiac Research Institute, Darlinghurst, NSW Australia; 6 Western Australian Register of Developmental Anomalies, King Edward Memorial Hospital, Subiaco, WA 6008 Australia; 7 Undiagnosed Diseases Program, Genetic Services of WA, Subiaco, Australia; 8 Present Address: Telethon Kids Institute, Northern Entrance, Perth Children’s Hospital, 15 Hospital Avenue, Nedlands, WA 6009 Australia

**Keywords:** Inducible pluripotent stem cells, CRISPR gene editing, Cardiac disease modelling

## Abstract

**Background:**

Genomic sequencing in congenital heart disease (CHD) patients often discovers novel genetic variants, which are classified as variants of uncertain significance (VUS). Functional analysis of each VUS is required in specialised laboratories, to determine whether the VUS is disease causative or not, leading to lengthy diagnostic delays. We investigated stem cell cardiac disease modelling and transcriptomics for the purpose of genetic variant classification using a GATA4 (p.Arg283Cys) VUS in a patient with CHD.

**Methods:**

We performed high efficiency CRISPR gene editing with homology directed repair in induced pluripotent stem cells (iPSCs), followed by rapid clonal selection with amplicon sequencing. Genetic variant and healthy matched control cells were compared using cardiomyocyte disease modelling and transcriptomics.

**Results:**

Genetic variant and healthy cardiomyocytes similarly expressed Troponin T (cTNNT), and GATA4. Transcriptomics analysis of cardiomyocyte differentiation identified changes consistent with the patient’s clinical human phenotype ontology terms. Further, transcriptomics revealed changes in calcium signalling, and cardiomyocyte adrenergic signalling in the variant cells. Functional testing demonstrated, altered action potentials in *GATA4* genetic variant cardiomyocytes were consistent with patient cardiac abnormalities.

**Conclusions:**

This work provides in vivo functional studies supportive of a damaging effect on the gene or gene product. Furthermore, we demonstrate the utility of iPSCs, CRISPR gene editing and cardiac disease modelling for genetic variant interpretation. The method can readily be applied to other genetic variants in *GATA4* or other genes in cardiac disease, providing a centralised assessment pathway for patient genetic variant interpretation.

**Supplementary Information:**

The online version contains supplementary material available at 10.1186/s13287-023-03592-1.

## Background

Congenital heart disease (CHD) is the most common, and often severe, anomaly at birth and affects 6–13 babies in every 1000 live births globally [1]. Recent studies indicate that over 400 genes may contribute to CHD [2], and these are often transcription factors that control cardiac cell differentiation [3, 4]. With the integration of genomics into patient care, an increasing number of novel and potentially disease-causing genetic variants are being identified. Novel variants without any prior disease association in another patient are classified as variants of unknown significance (VUS), in accordance with ACMG guidelines [5]. Each VUS will require validation in the laboratory to establish whether they are disease causative (pathogenic) or benign. This leads to major delays, with patients waiting on average 5 years, if not decades for diagnosis [6].

*GATA4* is a cardiac transcription factor with a highly conserved carboxyl Zn finger domain important for the formation of transcription factor complexes with NKX2.5, TBX5, and MEF2C [7, 8], mediating cardiac gene expression, including cardiac troponin T (cTNNT), and cardiac alpha-myosin heavy chain (MYH6) [9]. Accordingly, *GATA4* is important in the differentiation of cardiomyocytes; and known pathogenic variants in *GATA4* contribute to developmental heart defects including atrial septal defect, ventricular septal defect, atrioventricular septal defect, and tetralogy of Fallot [10, 11].

Recent advances in CRISPR homology directed repair (CRISPR_HDR) have improved gene editing efficiency from less than 1% to over 70% [12], in amenable laboratory cell lines [13]. This improved gene editing capacity in combination with the development of cardiac differentiation protocols provides an appropriate human model for the investigation of cardiac genetic variants.

In this study, we use inducible pluripotent stem cell (iPSC) gene editing, cardiac disease modelling, and transcriptomics for cardiac VUS classification. CRISPR_HDR is used to introduce a patient GATA4 VUS (p.Arg283Cys) into iPSCs (herein termed *GATA4*_HDR). *GATA4*_HDR and healthy control (*GATA4*_WT) iPSC were then differentiated into cardiomyocytes. The *GATA4*_HDR and *GATA4*_WT cardiomyocytes were compared at the cellular, transcriptomic, and functional levels to determine if changes were consistent with the patient’s phenotype.

## Methods

### Patient recruitment

Patient recruitment to this study was initiated by a genetic counsellor at Genome Services Western Australia, followed by written informed consent. The study adhered with the Declaration of Helsinki and the NHMRC National Statement on Ethical Conduct Human Ethics Research, and was approved by the Child and Adolescent Health Services, Human Research Ethics Committee, RGS0000000166.

### Patient genome sequencing

Target enrichment from Illumina TruSight One Expanded Kit, with massively parallel sequencing (Illumina NextSeq550), followed by secondary analysis with Illumina BWA Enrichment (v2.1.1), and tertiary analysis with Alissa Interpret (v5.1). The variant was classified as a Variant of unknown pathogenicity according to the ACMG guidelines [14].

### Cell culture

*HEK cell culture.* HEK293T cell lines were maintained in Complete Media (RPMI1640 (Gibco, Australia) with 10% Heat-Inactivated Foetal Bovine Serum (FCS) (CellSera, Australia), 1% sodium pyruvate (Gibco, Australia), 1% Penicillin/ Streptomycin Antibiotic (Gibco Australia), and 1% Glutamine (Gibco, Australia). Cells were dissociated with TryplE Express (Gibco, Australia). Cell cryopreservation was in Complete Media/10% DMSO.

*Stem cell culture*. KOLF2-C1 (KOLF2) cells were grown in 24-well plates coated with CellAdhere Vitronectin XF (STEMcell technologies), maintained in TeSR-E8 media (STEMCELL technologies), and media changed daily. Cells were split with Gentle Cell Dissociation Reagent with 10 µM ROCK Inhibitor (Y-27632, STEMCELL Technologies) for 24 h [13]. Cell cryopreservation was in CryoStor CS10 (STEMCELL Technologies).

All cultures were grown in a 37 °C humidified CO_2_ (5%) incubator, unless otherwise stated, and routinely checked for mycoplasma.

### CRISPR/Cas9 *GATA4*_HDR transfection and cloning

*HEK293T cell CRISPR_HDR transfection*. HEK293T cells were grown to 30–50% confluence, and CRISPR_HDR transfected with click chemistry [15]. Constructs were GATA4_Bg5_crRNA (5′ Dibenzocyclooctyne N-Hydroxysuccinimide, DBCON, AGACCACCACCACCACGCTG 3′, PAM: TGG; GATA4 NM_002052.4) and ssDNA GATA4_HDR_Bg5(+) (5′^AzideN^CTCCTGTGCCAACTGCCAGACCACCACCACTACACTCTGGTGCCGCAATGCGGAGGGCGAGCCTGTGTGCAATGCCTGCGG 3′). The GATA4_VUS HEK293T variant sequence mutalyzer description is NG_008177.2 (GATA4):g.78246_78264delinsTACACTCTGGTGTAGAAAC with affected protein NG_008177.2 (GATA4_i001):p. (Arg283Cys).

*iPSC CRISPR HDR transfection*. KOLF2 cells grown to 30–50% confluence, were dissociated with Gentle Cell Dissociation Reagent and 1 × 10^5^ cells in 400 µl mTeSR1 with 10 µM Y-27632 (STEMCELL technologies) aliquoted in 24 well plates. The 10 µM GATA4_AF_HDR, ssDNA (5′^Alt-R-HDR1^GTGGGCCTCTCCTGTGCCAACTGCCAGACCACCACCACCACGCTCTGGTGCCGCAATGCGGAGGGCGAGCCTGTGTGCAA^Alt-R-HDR2^ 3′), and 10 µM GATA4_AF crRNA (5′AGACCACCACCACCACGCTG 3′, PAM: TGG; GATA4 NM_002052.4) were purchased from Integrated DNA Technology (IDT). The gRNA was generated in Duplex Buffer (IDT, USA) with 1 µM crRNA (IDT), 1 µM tracrRNA-ATTO550 (IDT), heated to 95 °C for 5 min and cooled to RT. CRISPR ribonucleoprotein (RNP complexes) were formed with 63 nM gRNA and 63 nM high-fidelity Cas9 protein (IDT) in OPTIMEM, followed by addition of 63 nM HDR. RNPs were transfected into KOLF2 cells with STEM Lipofectamine (Life Technologies), according to the manufacturer’s instruction. Cultures were rendered 30 µM ALT-R HDR enhancer, incubated 48 h at 32 °C with a media change to remove Alt-R HDR Enhancer at 24 h. The introduced *GATA4*_HDR iPSC variant sequence mutalyzer description is NM_002052.4 (GATA4):c.843_847delinsCTGGT with affected protein NM_002052.4 (GATA4_i001):p. (Arg283Cys).

Following transfection cells were cultured at least 7 days prior to cell freeze down and genomic DNA extraction (PureLink™ Genomic DNA Mini Kit, Life Technologies). Percentage frequency of HDR in gDNA was determined by amplicon sequencing [13, 15, 16]. Transfected cells were single cell cloned by limiting dilution in 96 well plates, replica plated, and DNA lysate prepared [13, 15]. A second round of amplicon sequencing on DNA lysate determined clonal cell lines.

The top six off-target CRISPR crRNA gene cut sites were confirmed as WT by forward and reverse Sanger sequencing for HEK293 and KOLF2 cell clones (AGRF, WA; Additional file 2: Fig. S2A).

### Fluorescent immunohistochemistry

*GATA4*_WT and *GATA4*_HDR KOLF2 iPSC clones were cultured on Matrigel-coated chamber slides (ibidi) and differentiated into cardiomyocytes. Cardiomyocytes were fixed with 3.7% formaldehyde (Sigma-Aldrich) 20 min at room temperature and stored at 4 °C in DPBS. Subsequently, cells were permeabilised for 15 min with 0.1% Triton-X-100 (Sigma-Aldrich) and blocked with Intercept® Blocking Buffer (LI-COR) for 1 h at room temperature, incubated overnight at 4 °C with rabbit polyclonal anti-GATA4 antibody (1:400; D3A3M, Cell Signalling Technology), washed with 0.05% Tween20 (Sigma-Aldrich) and treated with Alexa-Fluor 488-conjugated anti-rabbit antibody (1:1000; Invitrogen) for 1 h at room temperature. Residual secondary antibody was removed by washing and the cell nuclei stained using NucBlue stain (Invitrogen). Antibody staining was visualised using a Nikon C^2+^ inverted confocal microscope with a Nikon 40× objective. Monochrome immunofluorescence images were captured and processed using NIS-Elements software (v.5.21.00) and Adobe Photoshop (v.22.3.1) to add colour and merge channels.

### Amplicon sequencing

Next-generation amplicon sequencing was carried out on the MiniSeq Sequencing System (Illumina^©^). In brief, a 250 bp *GATA4*_HDR site PCR product was amplified, from gDNA or DNA lysates, with *GATA4* pAMPF1 (^5′^ACACTCTTTCCCTACACGACGCTCTTCCGATCTcaccttttacttggacatgaagc^3′^) and *GATA4* pAMPR1 (^5′^GTGACTGGAGTTCAGACGTGTGCTCTTCCGATCTgtacaaaggaagaagacaaggga ^3′^) [16] for 150 bp, paired-end, > 10,000 reads (MiniSeq, Illumina, Australia) and reads aligned to the HDR or WT amplicon with CRISPResso2 software [17]. In addition, Sanger sequencing was performed across a GATA4 516 bp PCR product with GATA4_F1 primer ^5′^cggtcagttctcctctcagg^3′^ and GATA4_R1 primer ^5′^gagagatgggcatcagaagg^3′^ (AGRF, Perth, Western Australia).

### GATA4 Western Blot and Immunoprecipitation

Proteins were extracted from cells lysed with Pierce’s co-IP protein lysis buffer (ThermoFisher Scientific), quantified (Direct-Detect® Infrared Spectrometer, Merck Millipore), electrophoresed on NuPAGE Bis–Tris 4–12% protein gels (Life Technologies) and blotted onto Polyvinylidene fluoride (PVDF) membranes (Life Technologies). Membranes were blocked with Intercept® (TBS) Blocking Buffer (LI-COR® Biosciences) at 4 °C overnight, stained with rabbit anti-human GATA4 (1:1000, clone D3A3M, Cell Signalling Technology, Australia), and/or b-Actin (1:5000; MA5-15729; Life Technologies, Australia), with secondary stain with goat anti-rabbit IRDye® 800CW (1:10,000; LI-COR® Biosciences) or goat anti-mouse IRDye® 680RD (1:10,000; LI-COR® Biosciences) and imaged on the Odyssey® Infrared Imaging System (LI-COR® Biosciences).

*IP protocol.* Protein G Dynabeads (Invitrogen, Australia) were washed, and resuspended in 5 µg NKX2.5 antibody (clone F-2, Sana Cruz) or isotype IgG control (Sigma, Australia) in 200 µL PBS/0.02% Tween20, incubated 10 min at room temperature with rotation, placed on the DynaMag-2 magnet, and the supernatant removed. The bead-antibody complex was washed once in 200 µL PBS (0.02% Tween20), twice in 200 µL of Conjugation Buffer (20 mM Sodium Phosphate, 0.15 M NaCl pH 7–9), 250 µL of Pierce BS^3^ crosslinker (prepared in DMSO [Sigma-Aldrich, Australia]) added, and incubated 20 min at room temperature with rotation. The cross-linking was quenched with Quenching Buffer (1 M Tris HCl ph7.5) for 15 min at room temperature with rotation, washed three times in 200 µL of Li-Cor IP Lysis Buffer (Invitrogen, Australia), then 1 mL of sample lysate was added prior to incubation overnight at 4 °C with gentle rotation. Samples were then placed on the DynaMag-2 magnet (supernatant retained), and beads washed 3 times with PBS. Sample was eluted with 20 µL of Elution Buffer (50 mM Glycine pH2.8), then 7.5 µL of NuPAGE LDS Sample Buffer (Invitrogen, Australia), and 3 µL NuPAGE Reducing agent (Invitrogen, Australia) were added, and sample incubated 70 °C for 10 min prior to gel electrophoresis.

### Cardiomyocyte differentiation

KOLF2 iPSCs*,* genetic variant and normal, were stimulated to differentiate with STEMdiff Cardiomyocyte Differentiation and Maintenance Kit (STEMCELL, Vic, Australia). At day 0 and 20 of cardiomyocyte differentiation 5 × 10^5^ cells were collected, fix/permeabilised according to Transcription factor staining buffer set (eBioscience, US) and stained for expression markers for stem cells: OCT3/4 (OCT3/4-AF488, 1:20, clone 40/Oct-3 (RUO), BD Pharmingen) and NANOG (NANOG-BV421, 1:20, clone 16H3A48, BioLegend); and/or cardiomyocytes: GATA4 (GATA4-PE, 1:20, clone L97-56, BD Pharmingen) and cTNNT (cTnT-AF647, 1:20, clone 13-11, RUO, BD Pharmingen). Specifically, LIVEDEAD ef780 stain for 10 min (eBioscience, US), fix/permeabilised according to Transcription factor staining buffer set (eBioscience, US) and stained for stem cell expression markers (OCT3/4, NANOG), or stained with cardiac cell markers cTNNT and GATA4 (1:20, clone L97-56, BD Pharmingen). Samples were collected using an LSRII X20 flow cytometer (BD, Biosciences) with BD FACSDiva™ software (BD Biosciences), and analysed with FlowJo software (TreeStar Inc, Ashlan, OR, USA). Statistical analysis of data was with one-way ANOVA and Bonferroni’s correction for multiple testing performed using GraphPad Prism Version 8 software (GraphPad Software Inc., La Jolla, CA, USA).

In addition, cells were visualised by light microscopy, and representative video captured of beating cardiomyocytes. Beating cardiomyocytes were scored (1 – 5) according to percentage frequency beating cells: 1, ≥ 1%; 2, ≥ 20%; 3, ≥ 40%; 4, ≥ 60%; and 5, ≥ 80%.

### Assessment of intracellular calcium

Cells cultured in StemDiff Cardiomyocyte Maintenance Basal Media (STEMCELL) were exposed to Fluo-4 (5 µM, Life Technologies) for 20 min, followed by 20 min de-esterification step in Hepes-Buffered Solution (HBS) containing (in mM): 5.3 KCl, 0.4 MgSO_4_.7H_2_O, 139 NaCl, 1.8 CaCl_2_, 5.6 Na_2_HPO_4_.2H_2_O, 5 glucose, 20 Hepes and 2 glutamine (pH = 7.4 at 37 °C), not supplemented with Fluo-4. Fluo-4 fluorescence was then recorded in HBS at 37 °C in stream acquisition mode, recording 400 images at 10 ms intervals, using a Zyla 5.5 sCMOS camera attached to an inverted Nikon TE2000-U microscope (ex 480 nm, em 535 nm). MetaMorph® 7.10.3 was used to quantify the signal by manually tracing myocytes. An equivalent region not containing cells was used as background and subtracted. The signal for each cell (F) was normalised to basal signal (F_0_), yielding F/F_0_, therefore basal signal approximated 1.0. Frequency of spontaneous calcium transients were calculated as the number of transients over the total length of recording (4 s), FWHM (full width of half maximum) was calculated by fitting a Lorentzian function to the first calcium transient on each recording using GraphPad Prism9.4.1 software [18–20].

### RNA Sequencing

At day 0 and day 21 of cardiac differentiation cells were collected for RNA extraction (RNeasy Minikit, with DNase on column treatment; Qiagen). RNA integrity was determined on the bioanalyser (Australian Genomics Research Facility (AGRF), Perth, Western Australia). RNA sequencing was performed according to SureSelect Strand-Specific XT HS2 poly A RNA Library preparation for Illumina Multiplexed Sequencing, paired-end, 100 bp, 30 M reads on the NOVAseq 6000 platform (Illumina, USA) at Genomics WA (Perth, Australia).

### Data processing and alignment

We used UMItools [21] to de-duplicate our reads prior to alignment. All reads were aligned to the human genome (build GRCh38), using a modified version of the ENCODE ‘rna-seq-pipeline’ (https://github.com/ENCODE-DCC/rna-seq-pipeline), via the Cromwell wrapper software ‘caper’ (https://github.com/ENCODE-DCC/caper). Within the pipeline, reads were aligned to GRCh38 using the STAR aligner (v2.5.1b) [22] and known transcripts were quantified using Kallisto (v0.44.0) [23]. We performed basic QC analysis of mapped reads using SAMStat [24].

### Differential gene expression analysis

We imported estimated gene abundances into R 4.1.2 (https://www.R-project.org/) using the tximport function [25]. We applied the standard RNA-seq analysis using edgeR, limma and voom as previously described [26].

### Biological interpretation of sample differences

We used clusterProfiler [27], DOSE [28], and Camera [29] via the EGSEA wrapper [30] for gene set enrichment analysis. The full analysis script is reported in Additional file 1.

## Results

### Classification of a patient genetic variant GATA4_Arg283Cys as a VUS

The *GATA4* c.847C > T(p.Arg283Cys) genetic variant was identified during custom gene panel analysis and classified as a variant of unknown pathogenicity, according to ACMG guidelines [14] citing: a highly conserved arginine residue in the carboxyl zinc finger domain of the cardiac transcription factor GATA4 [10]; the variant has not been observed in control population databases; multiple in silico algorithms indicate pathogenicity; multiple missense variants at the same residue have been previously reported as pathogenic in the literature (p.Arg283Ala and p.Arg283Gln); however there was insufficient evidence to confirm the clinical significance. We therefore investigated how the introduction of this patient genetic variant would impact cardiomyocyte formation and function to provide in vivo functional studies supportive of a damaging effect on the gene or gene product.

### GATA4 patient phenotype and function

The patient’s phenotype was characterised using Human Phenotype Ontology (HPO) terms which included atrioventricular canal defect, abnormality of the mitral valve, muscular hypotonia, hypoglycaemia, and global developmental delay, amongst others (Additional file 2: Fig. S1A). In addition, we mapped umbrella terms for the daughter and parent HPO terms (Additional file 2: Fig. S1B).

The patient genetic variant *GATA4* c.[847C > T];[847 =] p.[(Arg283Cys)];[(Arg283 =)] lies in the carboxyl zinc finger domain (Additional file 2: Fig. S2A and B) of the protein which plays a role in protein–protein interaction. In cardiac cells, GATA4 functions by association with NKX2.5 and MEF2C, and in COS-1 cell gene expression studies missense genetic variants at the same residue, p.Arg283Ala and p.Arg283Gln, show reduced interaction with the NKX2.5 cofactor [31]. Therefore, we introduced the *GATA4* genetic variant into HEK293T cells creating a homozygous cell clone termed *GATA4*_VUS (Clone GATA4_B6; Additional file 2: Fig. S2C). However, no change in GATA4 protein expression or association with NKX2.5 was observed (Additional file 2: Fig. S2D, E). We hypothesised that the patient *GATA4*_VUS may require expression in cardiac tissue to affect cellular and molecular changes indicative of disease.

### Gene editing and derivation iPSCs that harbour the patient GATA4 genetic variant

Next, we introduced the variant into KOLF2 iPSCs using CRISPR_HDR for the purposes of cardiac disease modelling. After CRISPR_HDR transfection, *GATA4* VUS presence in cells was determined by amplicon sequencing [13, 15–17]. A high HDR rate of 32.8 ± 1.7% (mean ± SE) was achieved for the introduction of the patient *GATA4* genetic variant, with a NHEJ frequency of 7.8 ± 2.3%. Ninety-five single cell clones were derived by limiting dilution, and gDNA screened with high throughput *GATA4* amplicon sequencing [13, 15], and subsequently Sanger sequencing (Additional file 2: Fig. S3), to confirm the presence of the VUS and WT alleles in our edited cells [13, 15–17]. CRISPR off-target analysis confirmed gDNA sequence integrity at the top six off-target crRNA sites by Sanger sequencing (Additional file 2: Fig. S4). We selected three heterozygous iPSC clones (GATA4_HDR_2B2, *GATA4*_HDR_1D1, *GATA4*_HDR_3H1) herein termed *GATA4*_HDR, and three matched healthy *GATA4* wild-type clones (*GATA4*_WT_3G2, *GATA4*_WT_3F2, *GATA4*_WT_4D1).

These data demonstrate a rapid and high throughput approach for clonal selection of genetic variant iPSCs after CRISPR_HDR.

### *GATA4*_WT and *GATA4*_HDR iPSCs differentiate into cardiomyocytes

**Fig. 1:**
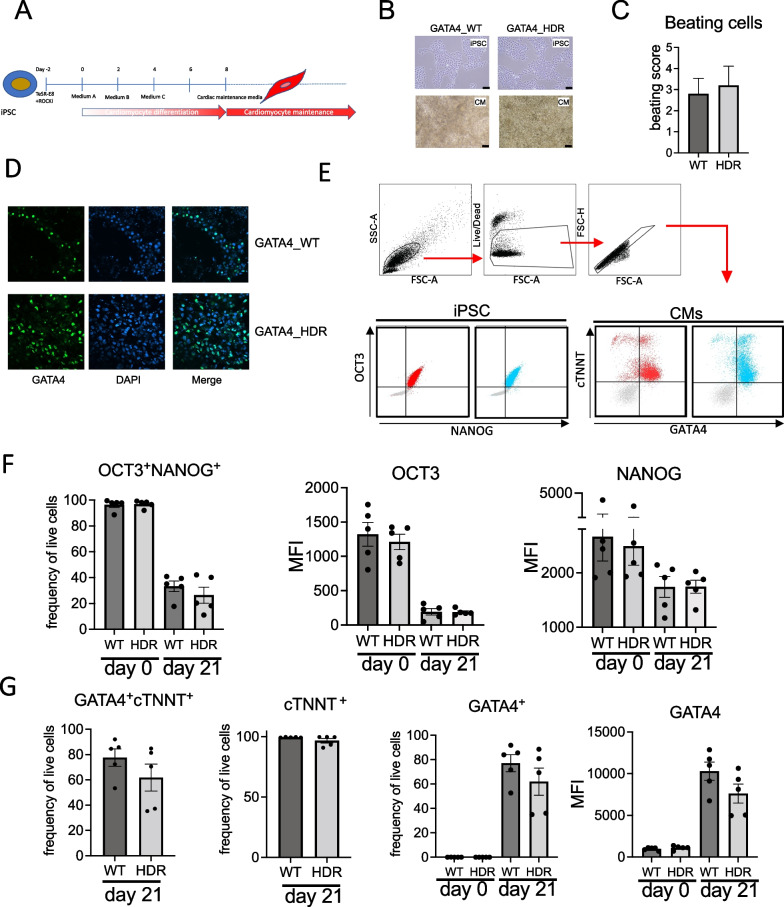
*GATA4*_WT and *GATA4*_HDR iPSC cardiac disease modelling. **A** iPSCs were subject to cardiomyocyte differentiation as indicated in the schematic. **B** Light microscopy images indicate cell morphology for iPSC and cardiomyocytes as indicated. **C** Histogram plot indicates beating scores for cardiomyocytes at day 20. **D** Fluorescent immunohistochemistry indicates that *GATA4*_WT and *GATA4*_HDR protein localises to the nucleus. **E** Flow cytometry gating strategy for iPSC and cardiomyocyte cell marker expression. Cells were gated on forward and side scatter, cell viability and for singles. Subsequently cells were gated for OCT3 and NANOG expression, or cTNNT and GATA4 expression. Dot plot indicates expression in *GATA4*_WT (red) and *GATA4*_HDR (blue), isotype control staining indicated in grey. **F** Bar plots indicate OCT3^+^NANOG^+^ percentage frequency expression, OCT3 MFI, and NANOG MFI in *GATA4*_WT and *GATA4*_HDR cells at day 0 and day 20. **G** Bar plots indicate expression percentage frequency for GATA4^+^cTNNT^+^, cTNNT^+^ cells, GATA4^+^ cells, and GATA4 MFI at indicated timepoints (*n* = 5 experiments with paired WT and HDR clones. *p ≤ 0.05, two-way ANOVA, with Bonferroni’s correction for multiple testing). MFI, mean fluorescent intensity

The *GATA4*_HDR, and matched healthy *GATA4*_WT iPSCs were induced for cardiac differentiation (Fig. 1A), and cells examined for beating cardiomyocytes, GATA4 cellular location, and cardiac cell expression markers.

Cardiomyocytes had similar morphology by light microscopy (Fig. 1B) and beating pattern as shown in representative videos (Additional file 4: Video S1. *GATA4*_WT and Additional file 5: Video S2. *GATA4*_HDR). There was no significant difference in the percentage of beating cardiomyocytes in *GATA4*_HDR cells compared with *GATA4*_WT cells (Fig. 1C). Immunohistochemistry confirmed expected GATA4 nuclear localisation in both GATA4_WT and GATA4_HDR cells (Fig. 1D).

To verify cardiomyocyte differentiation was successful we measured the expression of known cardiac and stem cell markers using flow cytometry. There were no significant differences in expression of stem cell markers, OCT3 and NANOG, or cardiac cell markers cTNNT and GATA4 at day 15 (data not shown) or day 21 (Fig. 1E–G) between *GATA4*_WT and *GATA4*_HDR cells.

### Transcriptomics identifies cardiomyocyte differentiation

**Fig. 2:**
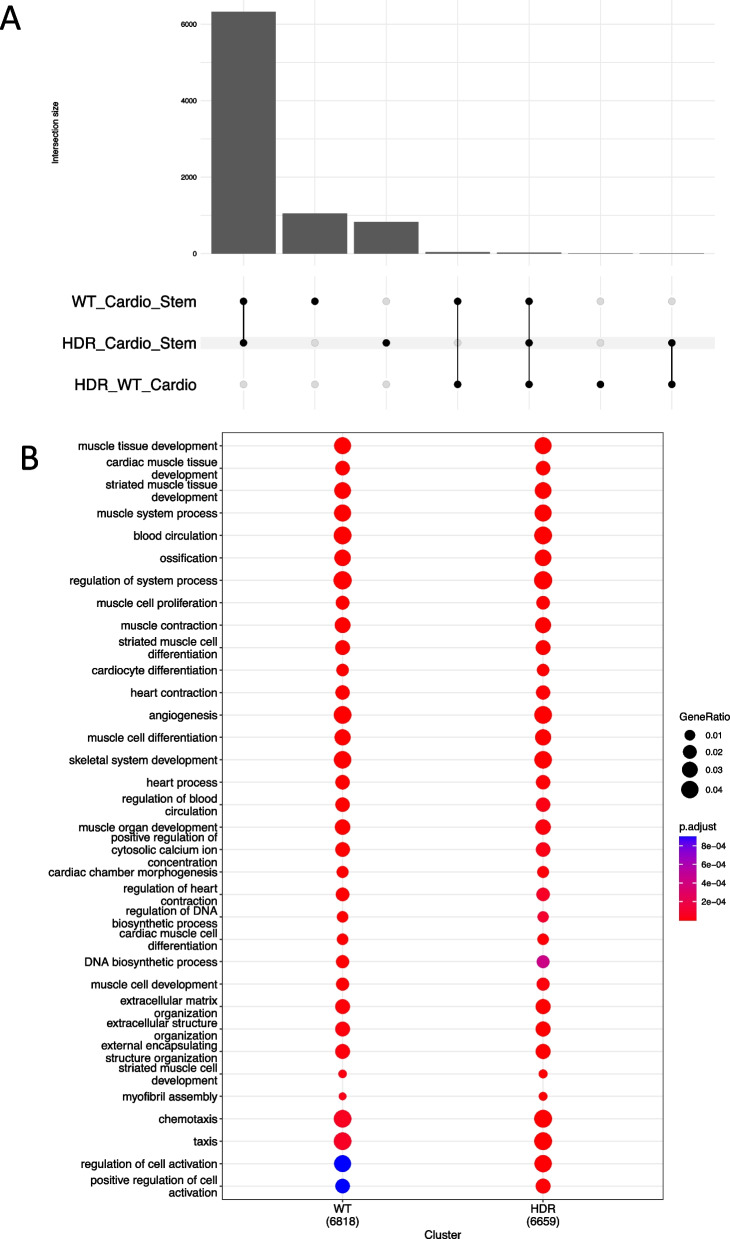
Changes in gene expression during iPSC cardiac differentiation of *GATA4*_WT and *GATA4*_HDR cells*.* iPSC clones for *GATA4*_WT and *GATA4*_HDR, were differentiated to cardiomyocytes and DEGs determined for the differences in expression during differentiation. **A** Upset plot indicate differences in gene expression between groups. **B** Significant genesets from GO terms for iPSC to cardiomyocyte differentiation for *GATA4*_WT and *GATA4*_HDR

Transcriptome sequencing was performed on iPSCs (day 0) and derived cardiomyocytes (day 21). Principal component analysis (PCA, Additional file 2: Fig. S5A) indicated clustering of iPSCs from cardiomyocytes for PC1, and separation of *GATA4*_WT and *GATA4*_HDR cardiomyocytes across PC2. We performed standard differential gene expression (DGE) analysis comparing iPSC and differentiated cells, and *GATA4*_WT and *GATA4*_HDR cardiomyocytes. While there are many differentially expressed genes when contrasting cardiomyocytes to iPSCs, only 71 genes were differentially expressed (adj. *p*-value < 0.05 and log fold change ≥ 0.5) when comparing *GATA4*_WT and *GATA4*_HDR differentiated cardiomyocytes (Fig. 2A; Additional file 3: Tables S1A–C).

To confirm that normal cardiac differentiation took place we interrogated the corresponding DGE lists using clusterProfiler [28] with biological process gene ontology terms (Fig. 2B). Our gene lists were highly enriched for terms associated with normal cardiomyocyte differentiation, including the term cardiocyte differentiation (adj. *p*-value 1.5e−7 in WT and 7.7e−7 in HDR; Fig. 2B; Additional file 3: Table S1D). We therefore concluded that the variant had no effect on the global differentiation trajectory.

### Comparative pathway changes in cardiac differentiation reflect the patient phenotype

To further explore the data, we performed gene set enrichment analysis (GSEA) using the DOSE package [28] in conjunction with gene ontology and DisGeNET collection on gene/disease associations [32]. We applied GSEA to the overall differences in gene expression between *GATA4*_WT and *GATA4*_HDR differentiation after adjusting for the mean expression at the differentiated cardiomyocyte stage by that at iPSC baseline. We discovered 373 disease terms enriched in our data (Additional file 3: Table S1E). Many of these terms were similar in their underlying gene lists.

**Fig. 3:**
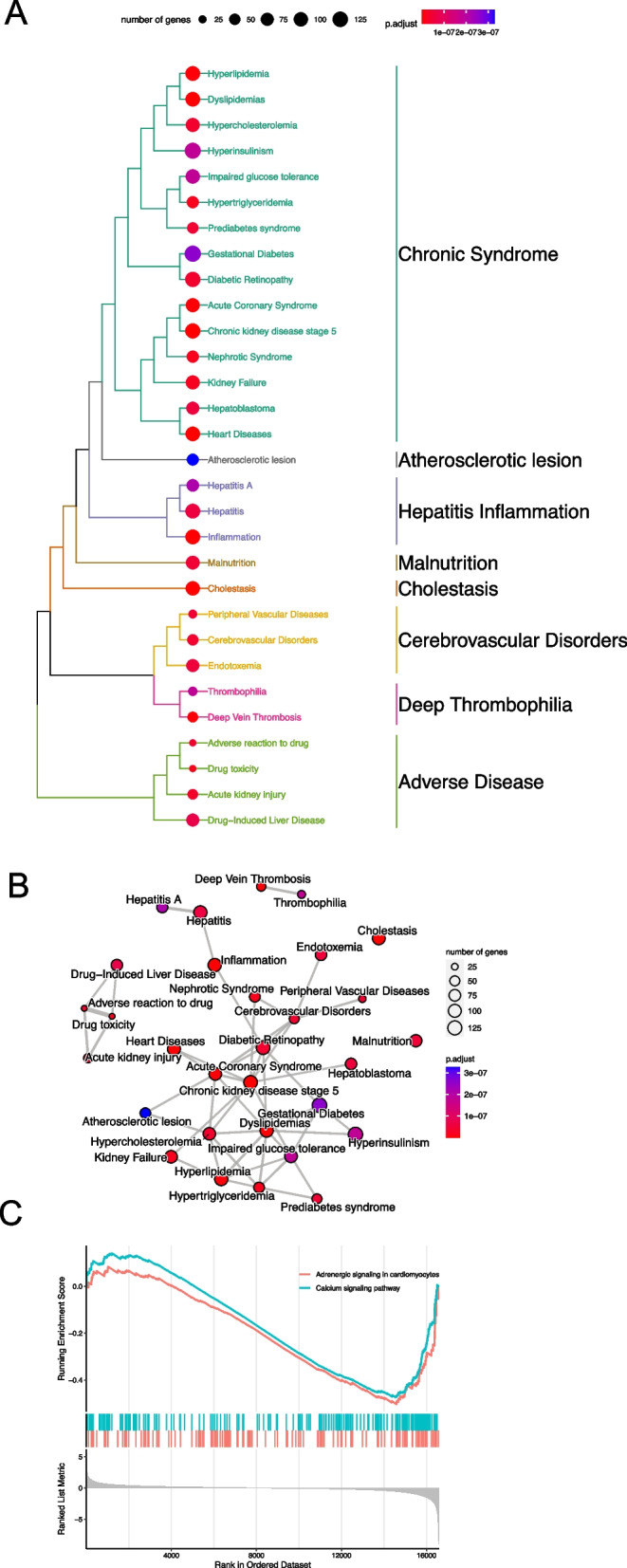
Difference in differentiation for *GATA4*_WT and *GATA4*_HDR reveals patient phenotype. **A** *GATA4*_HDR and G*ATA4*_WT differences in differentiation analysis with GSEA identifies significant disease pathways. **B** Network mapping and interaction for enriched pathways. **C** GSEA using KEGG pathways indicates calcium signalling and adrenergic signalling in cardiomyocytes is reduced in *GATA4*_HDR cell differentiation compared to *GATA4*_WT cell differentiation

To provide an overview we clustered terms by similarity (Fig. 3A) and determined gene network interactions (Fig. 3B and Additional file 2: Fig. S5B). Several terms were congruent with the patient’s phenotype. For example, the patient’s altered heart morphology (which is the parent term of Atrioventricular Canal Defect and Mitral Valve morphology, Additional file 2: Fig. S1) is reflected in the differential regulation of genes associated with heart disease. Likewise, disease terms associated with altered liver and kidney function were consistent with the patient’s hypoglycaemia and altered liver morphology. Finally, the presence of hepatic cysts, caused by malformations of the bile ducts, is consistent with the enrichment of genes implicated in cholestasis, a condition where bile cannot flow from the liver to the duodenum. Prior work has also identified GATA4 as a master regulator in liver development [33], more specifically hepatic microvascular specification and acquisition of organ specific vascular competence lending further support to our findings.

Additionally, we manually curated a list of terms with plausible associations with the patient’s cardiac disease phenotype from the list of 373 significantly enriched disease terms in DisGeNET enrichment (Additional file 2: Fig. S5C; Additional file 3: Table S1E). Terms specific to congenital heart defect and atrial septal defect symptoms included left ventricular hypertrophy, heart failure (chronic, left-sided, right sided), cardiac arrhythmia, tachycardia—ventricular, aortic valve stenosis, pulmonary hypertension, and congenital heart defects, amongst others [34]. Left ventricular hypertrophy is a known complication of AVSD, where additional blood flow from the vena cava causes increased total blood flow to the lungs, and subsequently increased pulmonary venous return via the left atrium to the left ventricle. Ultimately the increased blood flow to the lung causes right-sided heart failure, pulmonary hypertension of the arteries, and the increased volume to the left ventricle leads to hypertrophy and left-sided cardiac failure.

### Pathway, transcription factor binding, and functional changes in patient genetic variant cardiomyocytes

**Fig. 4:**
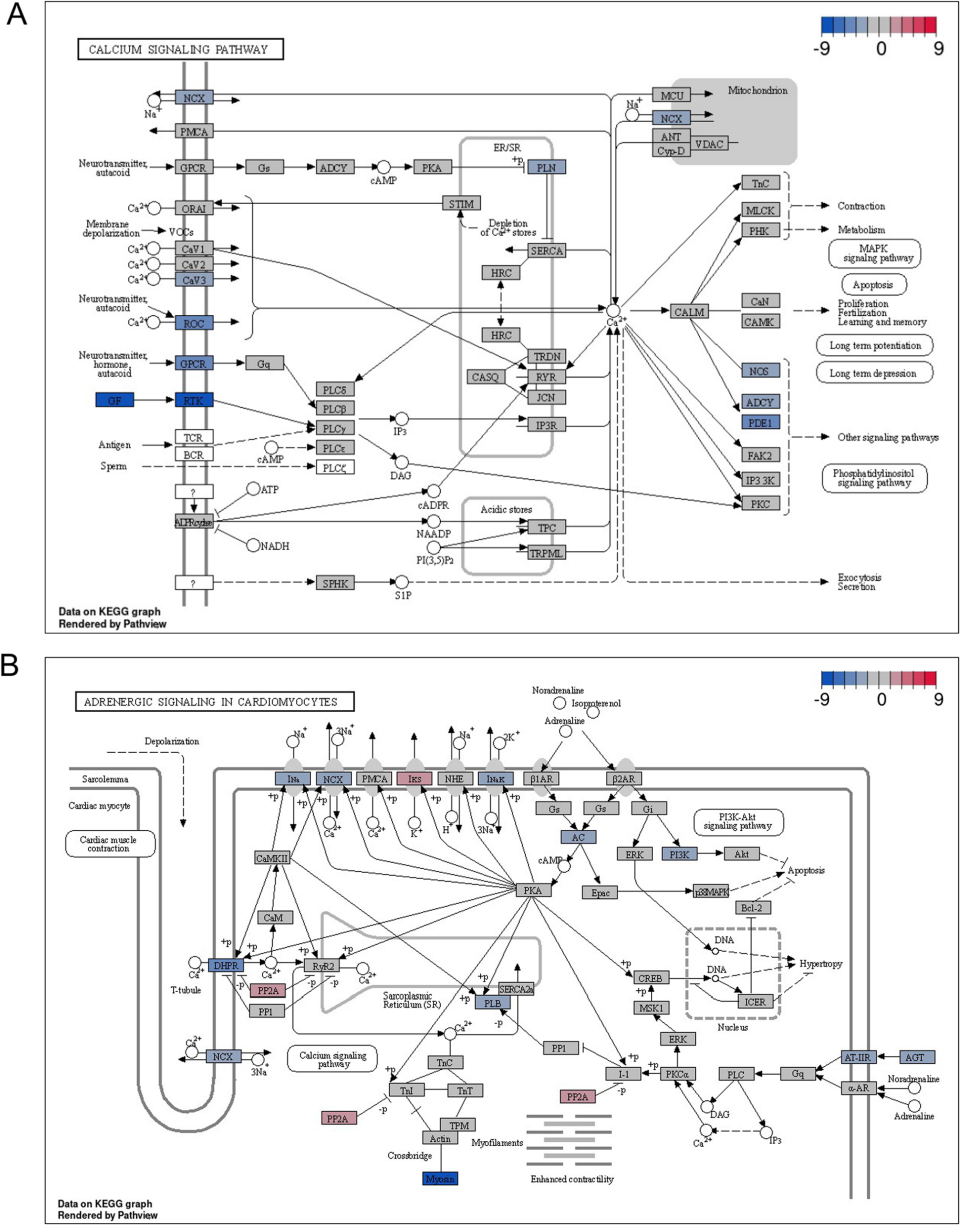
Difference in differentiation for *GATA4*_WT and *GATA4*_HDR reveals changes in calcium and adrenergic signalling pathways. **A** Differences in the differentiation of *GATA4*_HDR compared to *GATA4*_WT. Calcium signalling pathway changes as indicated **B**. Differences the differentiation of *GATA4*_HDR compared to *GATA4*_WT. Adrenergic signalling pathways in cardiomyocytes, as indicated

To better understand the biological pathways that may be involved in the differences observed in cardiac differentiation of *GATA4*_WT and *GATA4*_HDR we performed GSEA using KEGG pathways. Among the nine enriched pathways we discovered differential regulation in calcium signalling pathway and the related adrenergic signalling in cardiomyocytes pathway (Fig. 3C; Additional file 3: Table S1F). Genes in both pathways were upregulated during differentiation in both *GATA4*_WT and *GATA4*_HDR cells (Additional file 2: Fig. S6), with the upregulation of calcium signalling significantly lower in *GATA4*_HDR cells (adj. *p*-value < 0.035; Fig. 4).

Known ongoing complications of atrial septal defect include atrial tachyarrhythmia and atrial fibrillation [34]. Notably, genetic and familial studies indicate cardiac transcription factors including GATA4, TBX5, and NKX2.5 in atrial fibrillation pathogenesis [35, 36]. Atrial fibrillation is thought to develop due to changes mediated by early and delayed after depolarization (EAD, DAD) events, and is linked to changes in gene expression including the dysregulation of RYR2, reduced sarcoplasmic/endoplasmic reticulum Ca^2+^-ATPase2 (SERCA2, encoded by ATP2A2), and increased Na^+^/Ca^2+^ exchanger (NCX) [37]. GATA4 is reported to modulate RYR2 in human cells [37], and ATP2A2 expression in murine cardiac cells [37]. Further retinoic acid, an inducer of GATA4 transcription factor expression, increased NCX expression in rat cardiac atria cells [38]. The data in the patient like *GATA4*_HDR cardiomyocytes indicate reduced NCX gene expression, with milder changes in RYR2 expression and SERCA2, which may potentially be due to altered GATA4 regulation of gene expression. Potentially changes in the GATA4 p.Arg283Cys cells are due to the amino acid substitution in the zinc finger carboxyl domain that binds the major groove of DNA with the sequence element (A/T)GATA(A/G) leading to alterations in DNA binding and regulation of transcription (Additional file 2: Fig. S7).

Analysis using the EGSEA GenesetDB Gene Regulation pathways showed that the second most significant pathway down-regulated in GATA4_HDR cells was the FOXA2 transcription factor pathway (Adj *p* value = 3.37 × 10^−7^; Additional file 3: Table S1G). Interestingly, FOXA^2+^ mesoderm cells with increased GATA4 gene expression show enhanced differentiation to ventricular cardiomyocytes [39]. Also, in EGSEA Hallmark gene sets, HALLMARK_PI3K_AKT_MTOR_SIGNALLING (Genes upregulated by activation of the PI3K/AKT/mTOR pathway) was significantly altered in *GATA4*_HDR cells (adj *p* = 0.02456; Additional file 3: Table S1H). Interestingly in GATA4 p.G296S cardiomyocytes geneset enrichment of PI3K-Akt signalling has been previously indicated, with GATA4 providing a negative feedback loop to limited PI3K signalling [40]. This suggests similarities between these two GATA4 variant phenotypes.

**Fig. 5:**
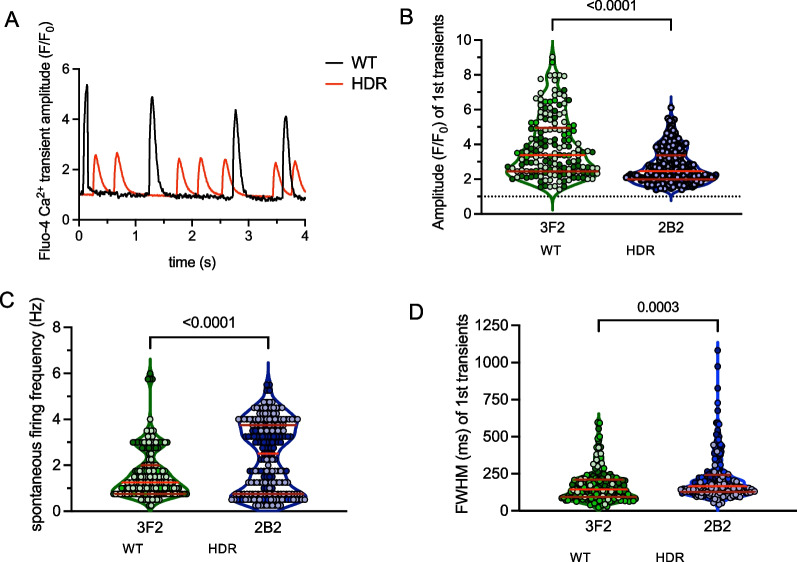
Difference in differentiation for *GATA4*_WT and *GATA4*_HDR reveals patient phenotype. **A** Graph indicates changes in calcium transients for *GATA4*_WT and *GATA4*_HDR cardiomyocytes. **B**–**D** Graphs indicate changes in Amplitude, spontaneous firing, and FWHM for *GATA4*_WT and *GATA4*_HDR cardiomyocytes (*n* = 3 experiments, Mann–Whitney test, *p* < 0.05)

To confirm our findings in calcium signalling, we compared calcium transients measured as Fluo-4 fluorescence in *GATA4*_HDR and *GATA4*_WT cardiomyocytes (Fig. 5). In *GATA4*_HDR cardiomyocytes the amplitude (F/F_0_) was significantly decreased (2.74 ± 0.07, and 3.86 ± 0.14, *p* < 0.0001). There was also increased spontaneous firing frequency (1/s) that was irregular (2.38 ± 0.10 Hz, and 1.64 ± 0.08 Hz, *p* < 0.0001) in the *GATA4*_HDR compared to *GATA4*_WT cardiomyocytes. Additionally, full width at half maximum (FWHM) was increased in *GATA4*_HDR compared to *GATA4*_WT cells (216.5 ± 10.3 ms and 171.4 ± 8.5 ms, *p* < 0.0003, respectively). These data indicate changes in cardiomyocyte calcium handling leading to increased and irregular contractability, with reduced excitability, in patient GATA4_HDR variant cardiomyocytes, consistent with the patient phenotype.

## Discussion

Rare disease diagnosis is often difficult and determining the pathogenicity of genetic variants of uncertain significance causes significant delays in patient diagnosis and treatment. In this study we investigate a VUS in the *GATA4* gene in a patient with CHD. CRISPR gene editing in iPSCs, cardiac disease modelling, and functional genomics provided evidence of pathogenicity for the variant that was supported by subsequent functional testing.

Application of CRISPR in iPSCs has improved due to new HDR methodology [12], and the availability of amenable iPSC lines [41]. In this study we applied high efficiency CRISPR_HDR and single cell clone selection with amplicon sequencing [13, 15] to generate a clonal population of iPSCs harbouring the GATA4 p.Arg283Cys patient genetic variant. Importantly, the derived iPSCs retained stem cell phenotype and were capable of differentiation to beating cardiomyocytes.

The genetic variant cardiomyocytes had similar morphology and cardiac marker expression of cTNNT and GATA4 compared to controls. Interestingly, however, using transcriptomics we were able to detect changes in gene expression during cardiac differentiation, where groups of known disease genes showed altered patterns in gene expression. This analysis identified geneset pathways consistent with the patient phenotype. Further, on a molecular level, we were able to identify pathways that were altered in the variant containing cells that were linked to both the heart and liver phenotype in the patient.

In transcriptomics pathway analysis for the differences in cardiomyocyte differentiation there were alterations in calcium signalling and adrenergic signalling. Studies have correlated changes in GATA4 expression with dysregulation of NCX1, RYR2, and SERCA2 expression, potentially due to GATA4 upstream dysregulation of gene expression in other models [37, 38]. Interestingly as indicated by others [40] we also observed upregulation of the PI3K-Akt pathway in GATA4 genetic variant cells, and this may provide a potential mechanism to restore normal function with PI3K inhibitors [40].

Calcium plays an important role in signalling pathways that direct heart development, in addition to excitation contraction coupling in cardiomyocytes [42]. The genetic variant GATA4 p.Arg283Cys cardiomyocytes demonstrated changes in the rate of calcium transients affecting cell contractility and excitability, indicative of heart dysfunction. There was an increased number of spontaneous calcium transients that were both smaller and wider, reflecting an increase in heart rate, weaker contractility, and irregular heart rate that is observed patient’s with CHD. The patient-like genetic variant *GATA4*_HDR cardiomyocytes show increased beat rate, but not strength because transient peaks are smaller than *GATA4*_WT control cardiomyocytes. Furthermore, *GATA4*_HDR cardiomyocyte beating rate was irregular, potentially reflecting an arrhythmia such as atrial fibrillation and/or a heart block (consistent with atrioventricular canal defect and abnormal mitral valve) [43].

Over 400 CHD related genes are known, and each gene harbours a spectrum of genetic variants that are currently classified as VUS. Pathogenic variants in the cardiac transcription factor GATA Binding Protein 4 (*GATA4)* are known to cause developmental heart defects including atrial septal defect, ventricular septal defect, atrioventricular septal defect, and tetralogy of Fallot [10, 11]. However, ClinVar lists 453 patient genetic variants in the *GATA4* gene alone, and of these only 23 are known pathogenic genetic variants and 204 are classified as VUS. Based on our study, we suggest that our approach is likely to be able to resolve the function for many other variants in *GATA4*, and other CHD related genes, which are currently classified as a VUS.

## Conclusion

The combination of CRISPR gene edited *GATA4*_HDR iPSCs with cardiac disease modelling and transcriptomics analysis provides supporting data towards disease pathogenicity for the patient GATA4 pArg283Cys VUS, that could not be determined with functional analysis in a standard laboratory cell line. The observed *GATA4*_HDR cardiomyocyte action potential changes were indicative of the patient heart disease phenotype. Finally, the study provides a disease appropriate model for the investigation of rare *GATA4* genetic variants in patients with cardiac disease, or CHD. Importantly, the methodology applied here is broadly applicable to the analysis of novel genetic variants identified in other genes relevant to cardiac disease.

### Supplementary Information

**Additional file 1**. GATA4 Project. Full analysis script for the study.

**Additional file 2: Figure S1** Human Phenotype Ontology mapping. **A** Petient HPO terms. **B** Human phenotype ontology tree indicating parent and daughter HPO terms. **Figure S2** GATA4 p.Arg283Cys interacts with cardiac transcription factor NKX2.5. **A** and **B** Protein structure of GATA4 indicating carboxy zinc finger domain (C’ Znf), and site Arginine to Cysteine mutation. **C** Introduction of GATA4pArg383Cys mutation in HEK293T cells and deep amplicon sequencing to determine genetic variant introduction in genomic DNA. **D** *GATA4*_WT (WT/WT), and homozygous *GATA4*_VUS (HDR/HDR) HEK293 cell protein expression for GATA4, and bactin by western blot. E, Immunoprecipitation of NKX2.5 in HEK293 *GATA4*_WT (WT/WT) and *GATA4*_VUS (HDR/HDR) indicated complex formation with GATA4. bactin expression in lysate preparation by western blot. **Figure S3** *GATA4*_WT and *GATA4*_HDR clonal genotypes. CRISPResso analysis of amplicon sequencing demonstrating WT and genetic variant allele expression. **A** Total reads per cell line amplicon. **B** Frequency of genetic variant and WT representation. **C** GATA4 cell clones were amplicon sequenced. FASTQ data file from CRISPResso analysis as indicated. **D** Sanger sequencing histograms of WT and genetic variant GATA4 clones as indicated. Solid blue arrow indicates patient C > T, p.Arg283Cys; dotted line indicates silent mutation G > C p.Leu281 = . **Figure S4** **A** Off-target sites and primer sequences. **B** Representative sanger sequencing at off-target sites. * Reverse complement sequence. **Figure S5** Global changes comparing, and contrasting, *GATA4*_WT and *GATA4*_HDR cells. **A** Principal component analysis indicating iPSCs and cardiomyocytes for *GATA4*_WT or *GATA4*_HDR cells. **B** Difference in differentiation gene network changes. **C** Additional cardiac terms in DisGenNet Enrichment for comparison of cardiac differentiation between *GATA4*_WT or *GATA4*_HDR. **Figure S6** Calcium and adrenergic signalling changes in cardiac differentiation. Gene set enrichment analysis using KEGG pathways. **A** *GATA4*_WT iPSC to cardiomyocyte differentiation. **B** *GATA4*_HDR iPSC to cardiomyocyte differentiation. **Figure S7** Altered Zn finger domain in GATA4 pArg283Cys protein.

**Additional file 3: Table S1A** Wild type comparing cardiomyocytes to stem (GeneLevelDE_diff_WT_Cardio_Stem). **Table S1B** HDR comparing cardiomyocytes to stem (GeneLevelDE_diff_HDR_Cardio_Stem). **Table S1C** Difference during differentiation (GeneLevelDE_diff_HDR_WT_Cardio_Stem). Gene set enrichment results. **Table S1D** GO term significantly enriched in wild type and HDR differentiation (GOTermEnrichedDifferentiation). **Table S1E** DisGenNet terms enriched in differences in differentiation comparison (DisGenNetEnrichedWT_HDR). **Table S1F** Kegg pathways enriched in differences in differentiation comparison (KEGGenrichedDifffWt_HDR). **DataS1G** Geneset DB regulation in differences in differentiation comparison (GenesetDBreg-DiffWT_HDR). **DataS1H** Geneset Hallmark in differences in differentiation comparison (GenesetHallmark-DiffWT_HDR).

**Additional file 4: Video S1** *GATA4*_WT cardiomyocytes.

**Additional file 5: Video S2** *GATA4*_HDR cardiomyocytes.

## Data Availability

Raw FASTQ files and processed count data for bulk RNA-seq are available at the Gene Expression Omnibus repository under accession number GSE229879. GATA4 amplicon sequencing files for clonal cell lines are also available. The Additional file 1 section provides all the code to reproduce the analysis and figures in this paper.

## Abbreviations

CRISPR: Clustered regularly interspaced short palindromic repeats
crRNA: Crispr RNA
DEG: Differentially expressed gene
gDNA: Genomic deoxyribonucleic acid
HDR: Homology directed repair
iPSC: Inducible pluripotent stem cells
NGS: Next-generation sequencing
CM: Cardiomyocytes
PAM: Protospacer adjacent motif
RNA: Ribonucleic acid
SNV: Single nucleotide variant
ssDNA: Single stranded DNA
TF: Transcription factor
tracrRNA: Trans-acting crRNA
WT: Wildtype
VUS: Variant of uncertain significance
