# Supplementary material for: Gene editing and cardiac disease modelling for the interpretation of genetic variants of uncertain significance in congenital heart disease
Source: Stem Cell Res Ther. 2023 Dec 5;14:345. doi: 10.1186/s13287-023-03592-1 (PMC10696868; doi:10.1186/s13287-023-03592-1)
Supplement: Supplementary file 1 — Additional file 1. GATA4 Project. Full analysis script for the study. [file 13287_2023_3592_MOESM1_ESM.pdf]

GATA4 Project

1 Introduction

This document contains all the code to reproduce the analysis figures for the paper:
" Functional analysis of genetic variants in GATA4 via stem cell cardiac disease modelling. "

2 Patient Phenotype plots

Here we generate a simple figure showing the patient HPO terms and their parent terms.

2.1 Step 1:

Download the latest HPO release:

```
datalad download-url -m 'Downloading GO obo file ' -O ../data/hp.obo http://purl.
    ↪ obolibrary.org/obo/hp.obo
```

Explanation of the format is here: http://owlcollab.github.io/oboformat/doc/GO.format.obo-1_2.html

2.2 Step 2

Load ontology and create plot:

```
library(ontologyIndex)
library(ontologyPlot)
setwd("../scratch")

hpo <- get_ontology("../data/hp.obo")
g <- onto_plot(hpo, terms=get_ancestors(hpo, c("HP:0001407",
"HP:0001943",
"HP:0001263",
"HP:0002019",
"HP:0002194",
"HP:0000527",
"HP:0002028",
"HP:0001252",
```

```
"HP:0000316",
"HP:0006695",
"HP:0001792",
"HP:0000750",
"HP:0001633",
"HP:0000324")));
write_dot(g, "OntologyTree.dot")
```

Here I performed some manual editing of dot file changing colors etc... Then:

```
dot -Tpdf OntologyTree.dot -o PatientHPO.pdf

cp PatientHPO.pdf ../results/PaperAnalysis
```

The corresponding legend should be:

Figure 1: Visualisations of the patient's phenotype described in human phenotype ontology terms.

3 Data Processing

The RNA-seq data was processed using a modified version of the ENCODE RNAseq pipeline. A copy of the corresponding wdl file is in the appendix.

We run the pipeline using slurm and singularity containers for complete reproducibility. The containers images are version controlled by git-annex via datalad[1].

Instead of using cromwell directly we use the caper / croo convenience scripts provided by the ENCODE-DCC. We wrote a small convenience script to create a python virtual environment with the software packages we need (see: Python virtual environment).

3.1 Configure caper / croo

```
./pipeline_config/capercroo_setup.sh
```

3.2 Prepare the sample sheet

Sample Number	Well ID	Sample Name	Other Comments	Pheno	ID	Repeat
1	A1	RNA 1	4D1 (1)	Stem	GATA4$_{4D1WTday0}$	1
2	B1	RNA 2	3H1 (1)	Stem	GATA4$_{3H1HDRday0}$	1
3	C1	RNA 3	4D1 (1)	Cardio	GATA4$_{4D1WTday20}$	1
4	D1	RNA 4	3H1 (1)	Cardio	GATA4$_{3H1HDRday20}$	1
5	E1	RNA 10	1D1 (1)	Stem	GATA4$_{1D1HDRday0}$	1
6	F1	RNA 15	3F2 (3)	Stem	GATA4r1$_{3F2WTday0}$	1
7	G1	RNA 16	2B2 (3)	Stem	GATA4r1$_{2B2HDRday0}$	1
8	H1	RNA 17	3F2 (3)	Cardio	GATA4r1$_{3F2WTday20}$	1
9	A2	RNA 18	2B2 (3)	Cardio	GATA421$_{2B2HDRday20}$	1
10	B2	RNA 19	3F2 (4)	Stem	GATA4r2$_{3F2WTday0}$	2
11	C2	RNA 20	2B2 (5)	Stem	GATA4r2$_{2B2HDRday0}$	2
12	D2	RNA 21	3F2 (4)	Cardio	GATA4r2$_{3F2WTday20}$	2
13	E2	RNA 22	2B2 (4)	Cardio	GATA4r2$_{2B2HDRday20}$	2
14	F2	RNA 23	3G2 (1)	Stem	GATA4$_{3G2WTday0}$	1
15	G2	RNA 24	3G2 (1)	Cardio	GATA4$_{3G2WTday20}$	1
16	H2	RNA 25	1D1 (1)	Cardio	GATA4$_{1D1HDRday20}$	1
17	A3	RNA 26	3F2 (2)	Stem	GATA4r3$_{3F2WTday0}$	3
18	B3	RNA 27	2B2 (2)	Stem	GATA4r3$_{2B2HDRday0}$	3
19	C3	RNA 28	3F2 (2)	Cardio	GATA4r3$_{3F2WTday20}$	3
20	D3	RNA 29	2B2 (2)	Cardio	GATA4r3$_{2B2HDRday20}$	3

```
library(tidyverse)
df = read_csv("../data/Sample_ann.csv")
df$`Sample Name` <- gsub(" ","-", df$`Sample Name`)
df$Sequencing_ID <- paste0(df$`Sample Name`,"_", df$ID,"_",df$Repeat)
x <- t(as.data.frame(str_split(df$ID,"_day")))
df$Day <- x[,2]
rm(x)
reformatted <- df %>% select(Sequencing_ID, Day, Pheno,Repeat  )
write_csv(reformatted, "../data/Sample_ann_reformatted.csv")
```

To create link the sample annotation with the corresponding fastq files, here we manually creates a csv file linking the two. For larger projects we use a script for the purpose. Here is the sample sheet we used:

```
"sample","library","readgroup","R1","R2"
"RNA-1_GATA4_4D1_WT_day0_1","GWA-VF-351","RG1","./data/GWA-VF-351/fastq/RNA-1_S1_L001_
    ↪ R1_001.fastq.gz","./data/GWA-VF-351/fastq/RNA-1_S1_L001_R2_001.fastq.gz"
```

```
"RNA-2_GATA4_3H1_HDR_day0_1","GWA-VF-351","RG1","./data/GWA-VF-351/fastq/RNA-2_S2_L001
    ↪ _R1_001.fastq.gz","./data/GWA-VF-351/fastq/RNA-2_S2_L001_R2_001.fastq.gz"
"RNA-3_GATA4_4D1_WT_day20_1","GWA-VF-351","RG1","./data/GWA-VF-351/fastq/RNA-3_S3_L001
    ↪ _R1_001.fastq.gz","./data/GWA-VF-351/fastq/RNA-3_S3_L001_R2_001.fastq.gz"
"RNA-4_GATA4_3H1_HDR_day20_1","GWA-VF-351","RG1","./data/GWA-VF-351/fastq/RNA-4_S4_
    ↪ L001_R1_001.fastq.gz","./data/GWA-VF-351/fastq/RNA-4_S4_L001_R2_001.fastq.gz"
"RNA-10_GATA4_1D1_HDR_day0_1","GWA-VF-351","RG1","./data/GWA-VF-351/fastq/RNA-10_S5_
    ↪ L001_R1_001.fastq.gz","./data/GWA-VF-351/fastq/RNA-10_S5_L001_R2_001.fastq.gz"
"RNA-15_GATA4r1_3F2_WT_day0_1","GWA-VF-351","RG1","./data/GWA-VF-351/fastq/RNA-15_S6_
    ↪ L001_R1_001.fastq.gz","./data/GWA-VF-351/fastq/RNA-15_S6_L001_R2_001.fastq.gz"
"RNA-16_GATA4r1_2B2_HDR_day0_1","GWA-VF-351","RG1","./data/GWA-VF-351/fastq/RNA-16_S7_
    ↪ L001_R1_001.fastq.gz","./data/GWA-VF-351/fastq/RNA-16_S7_L001_R2_001.fastq.gz"
"RNA-17_GATA4r1_3F2_WT_day20_1","GWA-VF-351","RG1","./data/GWA-VF-351/fastq/RNA-17_S8_
    ↪ L001_R1_001.fastq.gz","./data/GWA-VF-351/fastq/RNA-17_S8_L001_R2_001.fastq.gz"
"RNA-18_GATA421_2B2_HDR_day20_1","GWA-VF-351","RG1","./data/GWA-VF-351/fastq/RNA-18_S9
    ↪ _L001_R1_001.fastq.gz","./data/GWA-VF-351/fastq/RNA-18_S9_L001_R2_001.fastq.gz"
"RNA-19_GATA4r2_3F2_WT_day0_2","GWA-VF-351","RG1","./data/GWA-VF-351/fastq/RNA-19_S10_
    ↪ L001_R1_001.fastq.gz","./data/GWA-VF-351/fastq/RNA-19_S10_L001_R2_001.fastq.gz"
"RNA-20_GATA4r2_2B2_HDR_day0_2","GWA-VF-351","RG1","./data/GWA-VF-351/fastq/RNA-20_S11
    ↪ _L001_R1_001.fastq.gz","./data/GWA-VF-351/fastq/RNA-20_S11_L001_R2_001.fastq.gz
    ↪ "
"RNA-21_GATA4r2_3F2_WT_day20_2","GWA-VF-351","RG1","./data/GWA-VF-351/fastq/RNA-21_S12
    ↪ _L001_R1_001.fastq.gz","./data/GWA-VF-351/fastq/RNA-21_S12_L001_R2_001.fastq.gz
    ↪ "
"RNA-22_GATA4r2_2B2_HDR_day20_2","GWA-VF-351","RG1","./data/GWA-VF-351/fastq/RNA-22_
    ↪ S13_L001_R1_001.fastq.gz","./data/GWA-VF-351/fastq/RNA-22_S13_L001_R2_001.fastq.
    ↪ gz"
"RNA-23_GATA4_3G2_WT_day0_1","GWA-VF-351","RG1","./data/GWA-VF-351/fastq/RNA-23_S14_
    ↪ L001_R1_001.fastq.gz","./data/GWA-VF-351/fastq/RNA-23_S14_L001_R2_001.fastq.gz"
"RNA-24_GATA4_3G2_WT_day20_1","GWA-VF-351","RG1","./data/GWA-VF-351/fastq/RNA-24_S15_
    ↪ L001_R1_001.fastq.gz","./data/GWA-VF-351/fastq/RNA-24_S15_L001_R2_001.fastq.gz"
"RNA-25_GATA4_1D1_HDR_day20_1","GWA-VF-351","RG1","./data/GWA-VF-351/fastq/RNA-25_S16_
    ↪ L001_R1_001.fastq.gz","./data/GWA-VF-351/fastq/RNA-25_S16_L001_R2_001.fastq.gz"
"RNA-26_GATA4r3_3F2_WT_day0_3","GWA-VF-351","RG1","./data/GWA-VF-351/fastq/RNA-26_S17_
    ↪ L001_R1_001.fastq.gz","./data/GWA-VF-351/fastq/RNA-26_S17_L001_R2_001.fastq.gz"
"RNA-27_GATA4r3_2B2_HDR_day0_3","GWA-VF-351","RG1","./data/GWA-VF-351/fastq/RNA-27_S18
    ↪ _L001_R1_001.fastq.gz","./data/GWA-VF-351/fastq/RNA-27_S18_L001_R2_001.fastq.gz
    ↪ "
"RNA-28_GATA4r3_3F2_WT_day20_3","GWA-VF-351","RG1","./data/GWA-VF-351/fastq/RNA-28_S19
```

```
    ↪ _L001_R1_001.fastq.gz","./data/GWA-VF-351/fastq/RNA-28_S19_L001_R2_001.fastq.gz
    ↪ "
"RNA-29_GATA4r3_2B2_HDR_day20_3","GWA-VF-351","RG1","./data/GWA-VF-351/fastq/RNA-29_
    ↪ S20_L001_R1_001.fastq.gz","./data/GWA-VF-351/fastq/RNA-29_S20_L001_R2_001.fastq.
    ↪ gz"
```

The input `json` file used to run the pipeline is below. All reference files are version controlled using `git-annex` via `datalad`. Note that we included a UMI de-duplication step as out libraries contained a three nucleotide barcode followed by two random bases at the 5' end of the reads (pattern "`NNNXX`" below).

```
{
    "rnaseqcmpl.sampleConfigFile":"./scratch/SampleSheetGWA-VF-351.csv",
    "rnaseqcmpl.kallisto_index": "./references/assets/gencode.v39.transcripts_ercc_
    ↪ kallisto.idx",
    "rnaseqcmpl.star_index" : "./references/assets/STARv279a_GRCh38.primary_assembly_
    ↪ ERCC_v39_oh100.tar.gz",
    "rnaseqcmpl.genes_gtf" : "./references/assets/gencode.v39.genes.ERCC.gtf",
    "rnaseqcmpl.RSEMreference" : "./references/assets/rsem_reference_GRCh38_ERCC_v39.
    ↪ tar.gz",
    "rnaseqcmpl.somalierSiteList" : "./references/assets/sites.hg38.rna.vcf.gz",
    "rnaseqcmpl.umiDeduplication":  true,
    "rnaseqcmpl.variantCalling":  true,
    "rnaseqcmpl.bcPattern": "NNNXX",
    "rnaseqcmpl.bcPattern2": "NNNXX",
    "rnaseqcmpl.runname": "Test",
    "rnaseqcmpl.referenceFasta" : "./references/assets/GRCh38.primary_assembly_ERCC.
    ↪ fasta",
    "rnaseqcmpl.referenceFastaFai" : "./references/assets/GRCh38.primary_assembly_ERCC
    ↪ .fasta.fai",
    "rnaseqcmpl.referenceFastaDict" : "./references/assets/GRCh38.primary_assembly_
    ↪ ERCC.dict",
    "rnaseqcmpl.annotationsGTF" : "./references/assets/gencode.v39.annotation.ERCC.gtf
    ↪ ",
    "rnaseqcmpl.dbsnpVCF" : "./references/assets/Homo_sapiens_assembly38.dbsnp138.vcf"
    ↪ ,
    "rnaseqcmpl.dbsnpVCFIndex" : "./references/assets/Homo_sapiens_assembly38.dbsnp138
    ↪ .vcf.idx",
    "rnaseqcmpl.knownVcfs" : [
        "./references/assets/Homo_sapiens_assembly38.known_indels.vcf",
```

```
        "./references/assets/Mills_and_1000G_gold_standard.indels.hg38.vcf"] ,
    "rnaseqcmpl.knownVcfsIndices" : [
        "./references/assets/Homo_sapiens_assembly38.known_indels.vcf.idx",
        "./references/assets/Mills_and_1000G_gold_standard.indels.hg38.vcf.idx"]
}
```

To run the pipeline we ran the pipeline with the default configuration (see Cromwell config):

```
sbatch -J GATA4 --export=ALL --mem 5G --wrap "caper run -c pipeline_config/default.
  ↪ conf pipelines/rnaseq-cmpl.wdl -i ./scratch/GWA-VF-351.json --singularity -m ./
  ↪ scratch/GWA-VF-351_metadata.json --local-out-dir ./scratch --local-loc-dir ./
  ↪ scratch --cromwell-stdout ./scratch/cromwell.out"
```

Methods text:

3.3 TODO Methods text

4 Basic differential gene expression analysis [7/8]

These sections were run interactively, then we created corresponding Rscripts for each step.

4.1 Create metadata object in R

```
libraries <- c(
"tximport",
"tidyverse")
lapply(libraries, FUN = function(X) {
  do.call("library", list(X))
})

df = read_csv("../data/Sample_ann.csv")
df$`Sample Name` <- gsub(" ","-", df$`Sample Name`)
df$Sequencing_ID <- paste0(df$`Sample Name`,"_", df$ID,"_",df$Repeat)
df$CloneID <- gsub(" ([(][0-9][)])","", df$`Other Comments`)

add <- read_csv("../data/VF056RNAseqTable.csv")
add<- rename(add,"RNAname" = `RNA number`)

df$"Sample Name" = gsub("RNA-","",df$"Sample Name")
df <- rename(df ,"RNAname" = "Sample Name")
```

```
df$RNAname <- as.numeric(df$RNAname)

df <- inner_join(df, add, by = "RNAname")
df <-  df %>% select(-c("Sample Number", "Well ID","RNAname","Other Comments",`Stem/
    ↪ Cardio`,"Sample","Repeat"))

df <- df[order(df$CloneID),]

df <- df %>% relocate(ID, .after = CloneID)
df <- df %>% relocate(Sequencing_ID, .after = ID)
df <- df %>% relocate(CloneID, .before = Pheno)

df$CloneID <- as_factor(df$CloneID)
df$Pheno <- as_factor(df$Pheno)
## df$Day <- as_factor(df$Day)
## df$Repeat <- as_factor(df$Repeat)
df$Group <-  factor(grepl("HDR", df$ID), labels = c('WT','HDR') )
df <- df %>% relocate(Group, .before = CloneID)

files <- read_csv("sample_abundance_files.csv", col_names = FALSE)
colnames(files) = c("Path")
x <- gsub("../results/processing/Kallisto/","",files$Path)
files$Sequencing_ID <- gsub("/abundance.h5","",x)

SampleSheet  <- inner_join(x= df, y = files, by="Sequencing_ID")
rm(list=setdiff(ls(), c("SampleSheet")))
gc()
SampleSheet$Transfection <- as.factor(SampleSheet$Transfection)
SampleSheet$Differentiation <- as.factor(SampleSheet$Differentiation)
write_csv(SampleSheet,"SampleMetaData.csv")
```

4.2 Create annotation files

Below we will conduct both transcript and gene level analysis. Therefore we need to create two conversion tables, each taking gencode identifiers and converting them to entrez gene IDs or leving them as gencode transcripts identifiers.

4.2.1 Entrez table

The gencode project provides a gencode to entrez ID conversion table for every release. Let's download this file:

```
datalad download-url -m 'gencode.v39.metadata.EntrezGene.gz' -O ../data/gencode.v39.
    ↪ metadata.EntrezGene.gz \       https://ftp.ebi.ac.uk/pub/databases/gencode/
    ↪ Gencode_human/release_39/gencode.v39.metadata.EntrezGene.gz
```

And covert into a format `tximport` can use:

```
zcat ../data/gencode.v39.metadata.EntrezGene.gz | awk '{printf "%s,%s\n", $1, $2}'  >
    ↪ tx2gene_entrez.csv
```

4.2.2 Construct gene to transcript table from h5 file

This script reads in the list of paths to Kallisto h5 files from above, reads in one file and writes out a gene to expression table. NOTE: this code will only work when using gencode annotations, which I like because all the transcript and various gene names are linked to individual sequences.

We ended up using the Entrez based analysis so this part was actually not used to produce the results for the paper.

```
libraries <- c(
"tximport",
"tidyverse")
lapply(libraries, FUN = function(X) {
  do.call("library", list(X))
})

files <- read_csv("sample_abundance_files.csv", col_names = FALSE)
colnames(files) = c("Path")
x <- gsub("../results/processing/Kallisto/","",files$Path)
files$Sequencing_ID <- gsub("/abundance.h5","",x)

RNA <- tximport (files$Path[1] , type ="kallisto",txOut = TRUE)

df = data.frame(names  = rownames(RNA$abundance))
write_csv(df , "tmp.csv",col_names = FALSE)

cmd <- paste(
   "cat tmp.csv | awk '{ ",
    "n = split($0,a,\"|\");",
```

```
  "tx = a[1];",
  "if(n > 1){ ",
       "gene=a[6]; ",
    "}else{",
       "gene=a[1];",
    "}",
     "printf \"%s,%s\\n\",tx,gene; ",
     " }' > tx2gene_gencode.csv "
  )

system( cmd)
```

4.3 Basic QC analysis

4.3.1 Load data

We begin by loading the kallisto abundance files into R. We will use tximport[2] together with the entrez ID's from above.

First let's load the analysis packages:

```
libraries <- c(
 "clusterProfiler",
  "org.Hs.eg.db",
  "BiocParallel",
  "tidyverse",
  "RColorBrewer",
  "limma",
  "edgeR",
  "EGSEA",
  "tximport",
  "DRIMSeq",
  "stageR",
  "GGally",
  "ggcorrplot",
  "ggdendro",
  "ggpubr",
  "ggbeeswarm",
  "xlsx",
  "ComplexUpset",
  "DOSE",
```

```
  "enrichplot"
)

lapply(libraries, FUN = function(X) {
  do.call("library", list(X))
})
setwd("../scratch")
```

Load the data using the sample sheet created above.

```
meta <- read_csv("SampleMetaData.csv")
tx2gene <- read.csv("tx2gene_entrez.csv", header = FALSE)
txi <- tximport(meta$Path, type = "kallisto", tx2gene = tx2gene ,countsFromAbundance =
    ↪ "lengthScaledTPM",ignoreAfterBar = TRUE)
```

(Note the `SampleMetaData.csv` file is the sample sheet with an additional `Path` column linking each sample to the corresponding abundance file.)

4.3.2 Create DGE object:

From this point on the analysis replicates the standard voom / limma analysis[3].

```
y <- DGEList(txi$counts)
#Add meta data
meta$mGroup <- paste0(meta$Group,meta$Pheno)
y$samples$group = as.factor(meta$mGroup)
y$samples$Transfection = as.factor(meta$Transfection)
y$samples$Differentiation = as.factor(meta$Differentiation)
y$samples$CloneID = as.factor(meta$CloneID)
```

Add gene symbols:

```
x <- org.Hs.egSYMBOL
# Get the gene symbol that are mapped to an entrez gene identifiers
mapped_genes <- mappedkeys(x)
# Convert to a list
id_to_symbol_map  = as.data.frame(x[mapped_genes])

genes <-  id_to_symbol_map[id_to_symbol_map$gene_id %in% rownames(y$counts) ,]
colnames(genes) = c("FeatureID","Symbols")
y$genes = genes
x <- y
```

Cleaning up:

```
rm(list=setdiff(ls(), c("x")))
```

Save annotated DGE object:

```
saveRDS(file = "RAW_DGE.rds",  object = x)
```

4.3.3 Date pre-processing for visualisation:

```
x <- readRDS(file =  "RAW_DGE.rds")

cpm <- cpm(x)
lcpm <- cpm(x, log=TRUE)

L <- mean(x$samples$lib.size) * 1e-6
M <- median(x$samples$lib.size) * 1e-6
c(L, M)
```

Data filtering using the `filterByExpr` function from edgeR:

```
keep.exprs <- filterByExpr(x, group= x$samples$group)
x_filtered  <- x[keep.exprs, keep.lib.sizes=FALSE]
dim(x_filtered)
```

Plot filtered and unfiltered data. I will only plot the densities of the first 12 samples.

```
lcpm.cutoff <- log2(10/M + 2/L)
nsamples <- ncol(x)
if(nsamples > 12){
  nsamples <- 12
  }
col <- brewer.pal(nsamples, "Paired")
samplenames <- x$samples$group
```

```
par(mfrow=c(1,2))
lcpm <- cpm(x, log=TRUE)
plot(density(lcpm[,1]), col=col[1], lwd=2, ylim=c(0,0.26), las=2, main="", xlab="")
title(main="A. Raw data", xlab="Log-cpm")
abline(v=lcpm.cutoff, lty=3)
for (i in 2:nsamples){
  den <- density(lcpm[,i])
  lines(den$x, den$y, col=col[i], lwd=2)
}
```

```
lcpm <- cpm(x_filtered, log=TRUE)
plot(density(lcpm[,1]), col=col[1], lwd=2, ylim=c(0,0.26), las=2, main="", xlab="")
title(main="B. Filtered data", xlab="Log-cpm")
abline(v=lcpm.cutoff, lty=3)
for (i in 2:nsamples){
  den <- density(lcpm[,i])
  lines(den$x, den$y, col=col[i], lwd=2)
}
```

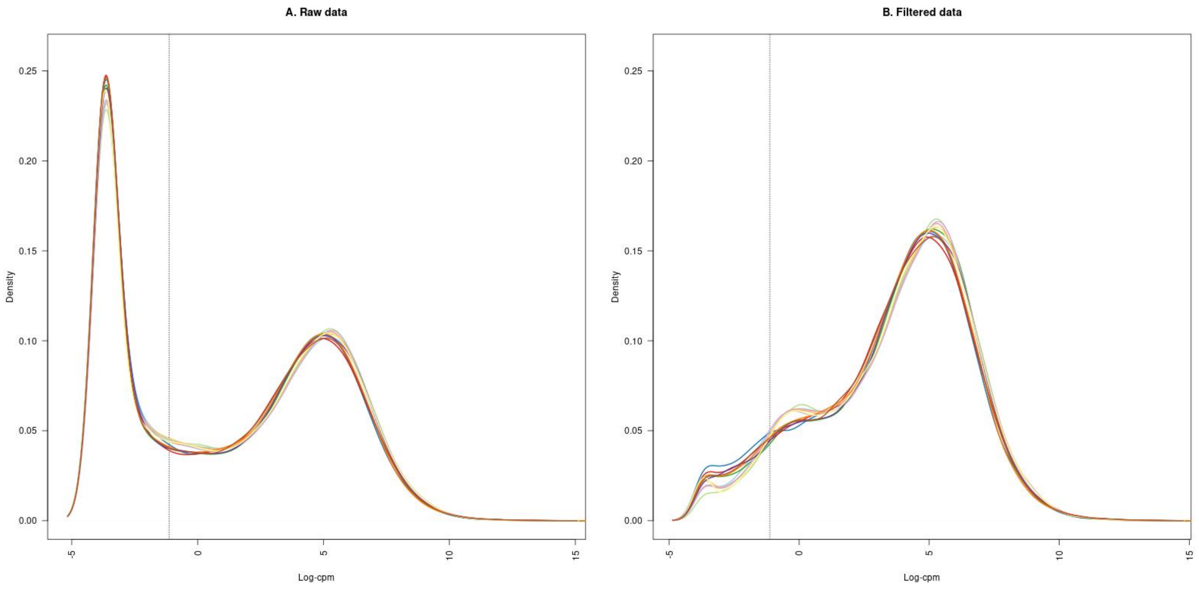

The above goes in the supplement.

4.3.4 Filter the gene expression matrix

Let's go ahead with the filtered data:

```
keep.exprs <- filterByExpr(x, group= x$samples$group)
x <- x[keep.exprs, keep.lib.sizes=FALSE]
dim(x)
```

4.3.5 Normalise data:

```
x1 <- calcNormFactors(x, method = "TMM")
x1$samples$norm.factors
```

Plot normalised vs not normalized data:

```
par(mfrow=c(1,2))
lcpm <- cpm(x, log=TRUE)
boxplot(lcpm, las=2, col=col, main="")
title(main="A. Example: Unnormalised data",ylab="Log-cpm")
lcpm <- cpm(x1, log=TRUE)
boxplot(lcpm, las=2, col=col, main="")
title(main="B. Example: Normalised data",ylab="Log-cpm")
```

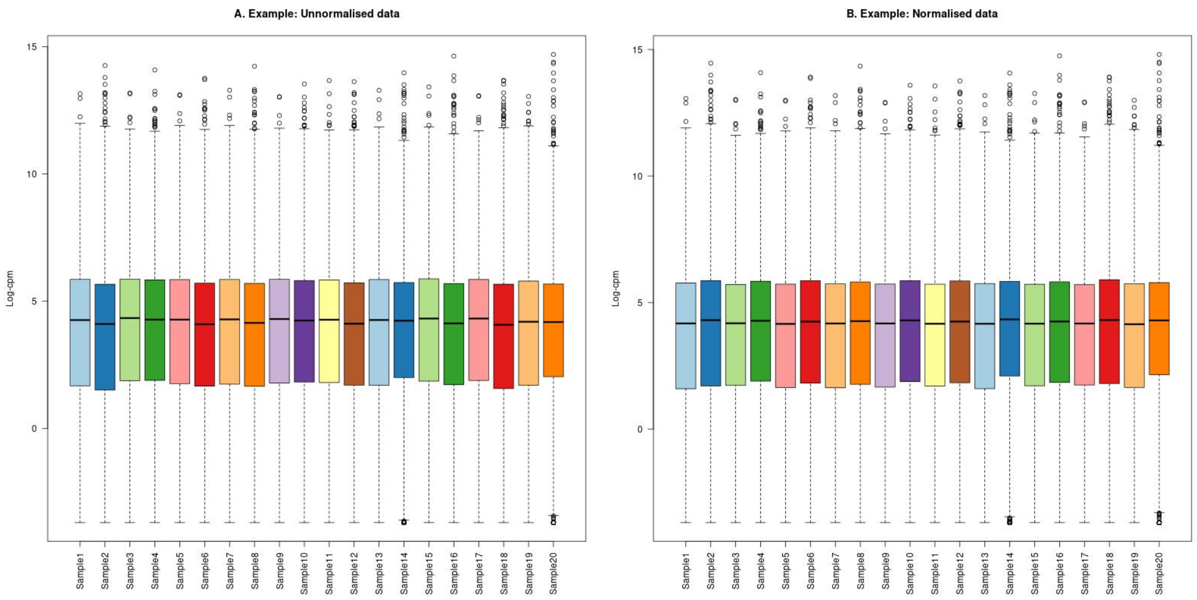

Goes in the supplement.

Looks good. Let's go ahead with the normalised data.

```
x <- x1
rm(list=setdiff(ls(), c("x")))
```

4.3.6 MDS plots of the samples:

```
lcpm <- cpm(x, log=TRUE)
par(mfrow=c(2,2))
col.group <- x$samples$group
levels(col.group) <-  brewer.pal(nlevels(col.group), "Set1")
col.group <- as.character(col.group)
```

```r
col.transfection <- x$samples$Transfection
levels(col.transfection) <-  brewer.pal(nlevels(col.transfection), "Set1")
col.transfection <- as.character(col.transfection)

col.differentiation <- x$samples$Differentiation
levels(col.differentiation) <-  brewer.pal(nlevels(col.differentiation), "Set1")
col.differentiation <- as.character(col.differentiation)

col.clone <- x$samples$CloneID
levels(col.clone) <-  brewer.pal(nlevels(col.clone), "Set2")
col.clone <- as.character(col.clone)


plotMDS(lcpm, labels=x$samples$group, col=col.group)
title(main="A. Groups")
plotMDS(lcpm, labels=x$samples$Transfection, col=col.transfection)
title(main="B. Transfection")
plotMDS(lcpm, labels=x$samples$Differentiation, col=col.differentiation)
title(main="C. Differentiation")
plotMDS(lcpm, labels=x$samples$CloneID, col=col.clone)
title(main="D. Clone ")
```

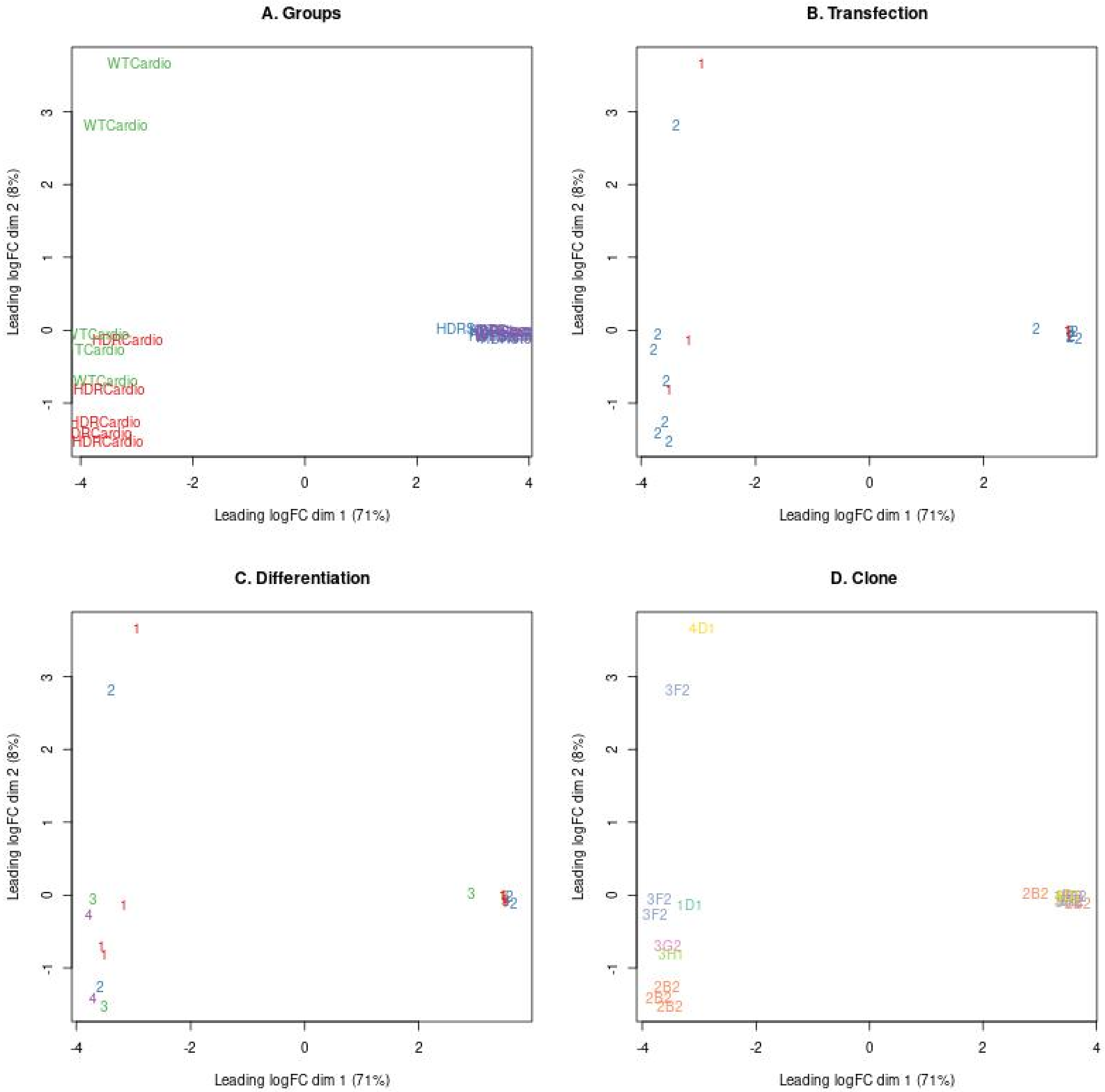

Produce publication quality MS plot.

```
lcpm <- cpm(x, log=TRUE)
par(mfrow=c(1,1))
col.group <- x$samples$group
levels(col.group) <-  brewer.pal(nlevels(col.group), "Set1")
col.group <- as.character(col.group)
pdf(file="../scratch/QC_MDS_group.pdf")
plotMDS(lcpm, labels=x$samples$group, col=col.group)
```

```r
dev.off()
```

Just to have a copy in this report:

```r
plotMDS(lcpm, labels=x$samples$group, col=col.group)
```

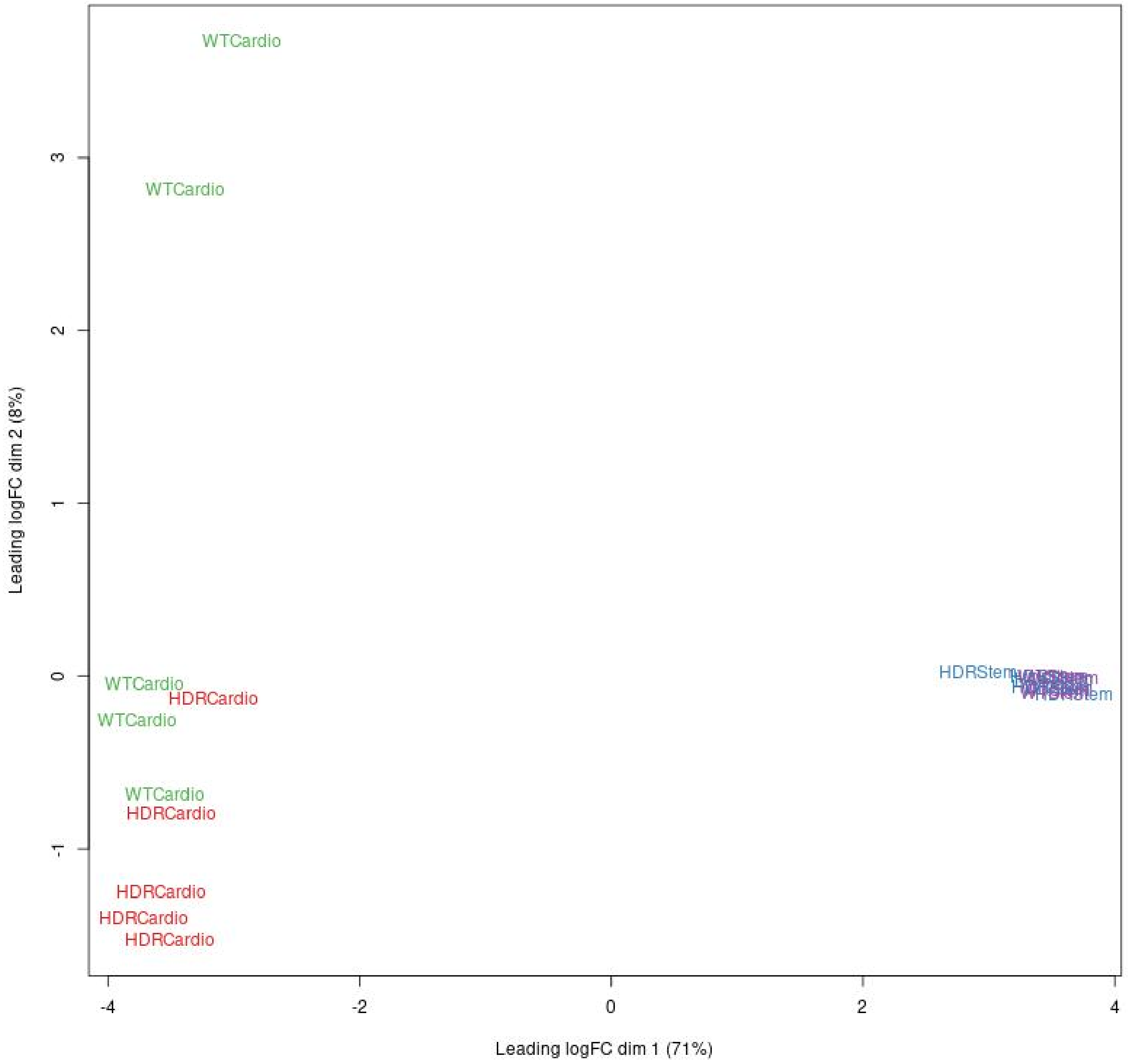

Figure X: Multidimensional scaling plot of distances between gene expression profiles. Distances on this plot reflect the typical log2 fold changes between the samples. Differences between iPSC and

cardiomyocyte samples account for 71.4% of variance explained while differences between edited and wild type samples account for 8.4%.

Ok looks good.

```
saveRDS(file = "NORM_DGE.rds",  object = x)
```

4.4 DE gene analysis

First let's load the analysis packages:

```
libraries <- c(
 "clusterProfiler",
  "org.Hs.eg.db",
  "BiocParallel",
  "tidyverse",
  "RColorBrewer",
  "limma",
  "edgeR",
  "EGSEA",
  "tximport",
  "DRIMSeq",
  "stageR",
  "GGally",
  "ggcorrplot",
  "ggdendro",
  "ggpubr",
  "ggbeeswarm",
  "xlsx",
  "ComplexUpset",
  "DOSE",
  "enrichplot"
)

lapply(libraries, FUN = function(X) {
  do.call("library", list(X))
})
setwd("../scratch")
```

Start in a clean state

```
rm(list=ls())
```

Read in normalised data and set design.

```
setwd('../scratch')
x <- readRDS("NORM_DGE.rds")
design <- stats::model.matrix(data= x$samples, ~0 + group + Transfection +
    ↪ Differentiation)
colnames(design) <- gsub("group", "", colnames(design))
```

Create voom object

```
v <- voom(x, design, plot=TRUE)
```

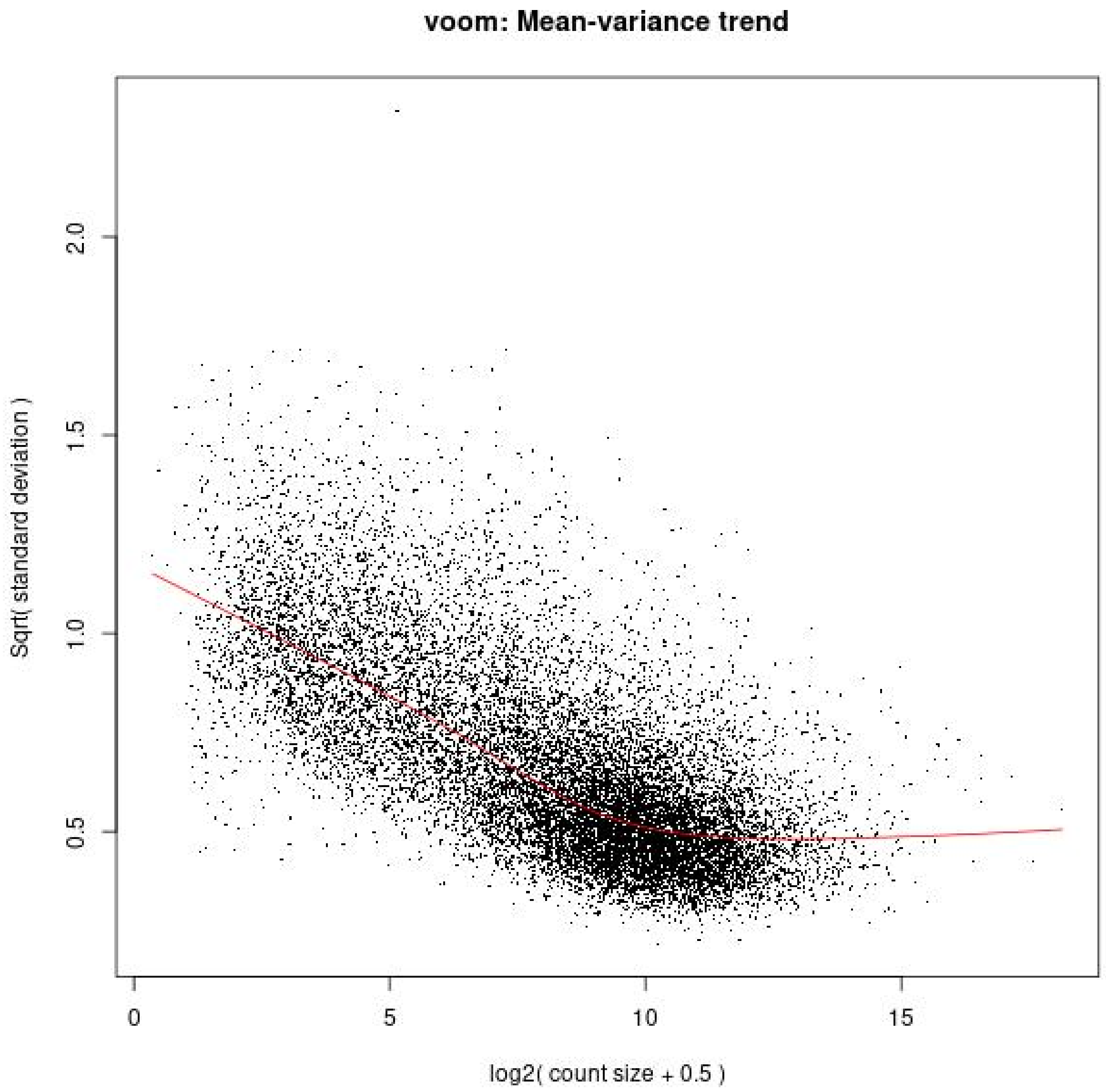

Define contrasts:

```
contr.matrix <- makeContrasts(
  diff_WT_Cardio_Stem = "WTCardio - WTStem",
  diff_HDR_Cardio_Stem = "HDRCardio - HDRStem",
  diff_HDR_WT_Stem = "HDRStem - WTStem",
  diff_HDR_WT_Cardio = "HDRCardio - WTCardio",
  diff_HDR_WT_Cardio_Stem = "(HDRCardio - HDRStem) - (WTCardio- WTStem)",
  levels = colnames(design))
```

Fit:

```
vfit <- lmFit(v, design)
vfit <- contrasts.fit(vfit, contrasts=contr.matrix)
efit <- eBayes(vfit)
fit <- treat(vfit, lfc=0.5)
```

Visualise the fit

```
plotSA(fit, main="Final model: Mean-variance trend")
```

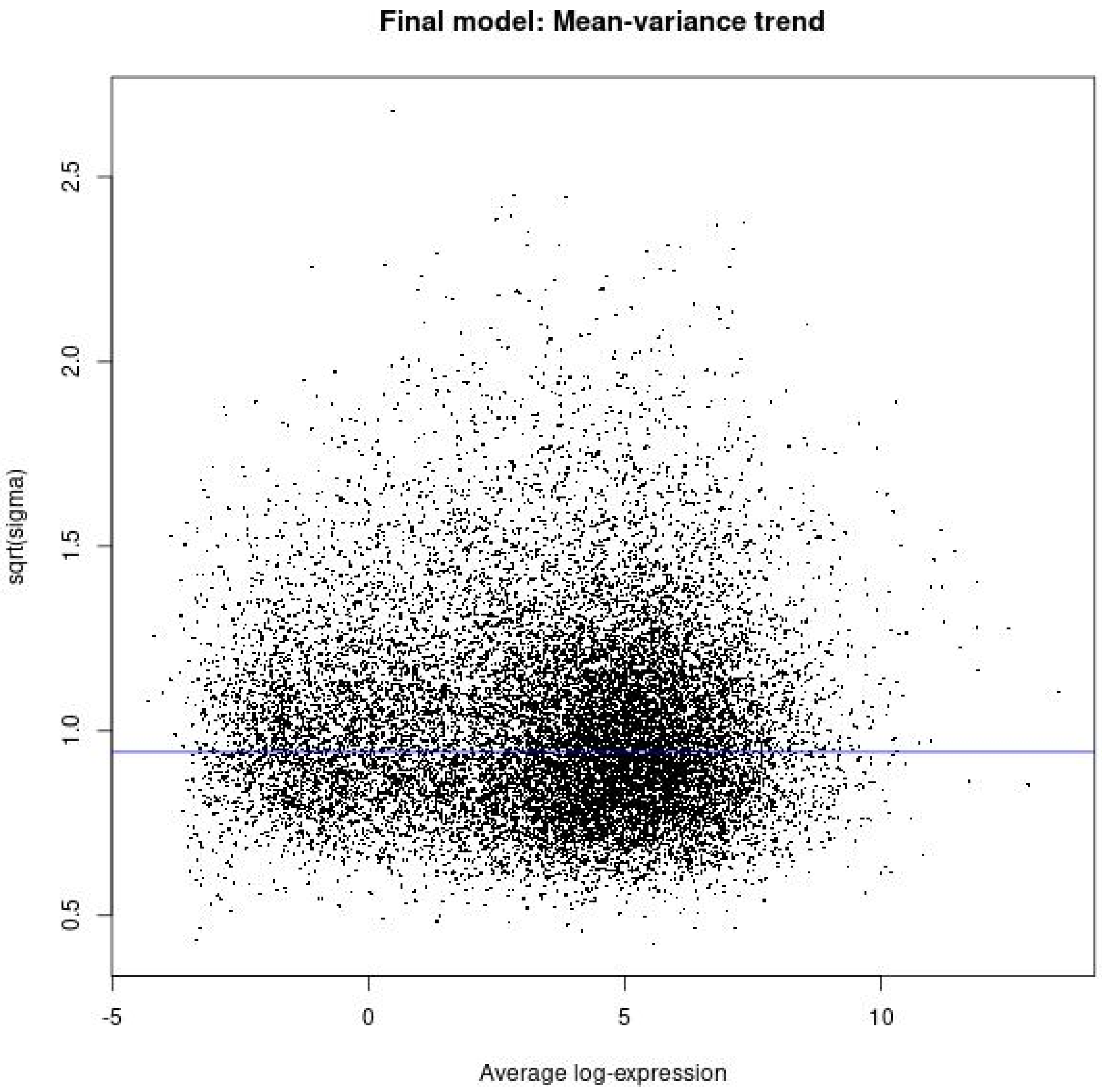

Number of differentially expressed genes in each contrast:

```
summary(decideTests(fit))
```

There are >6000 genes differentially expressed during differentiation but few to no genes different between WT and HDR samples.

Let's summarise these results in an upset plot:

Summarise DE results in upset plot:

```
dt <- decideTests(fit)
genres = colnames(dt)
x <- as.data.frame(dt)
x <- (replace(x, x == -1 , 1))
keep  <- (rowSums(x) != 0 )
x = x[keep,]
keep  <- (colSums(x) != 0 )
x = x[,keep]
genres = colnames(x)
DEgenesUpset <- upset(x, genres, name='Comparison' )
```

```
DEgenesUpset
```

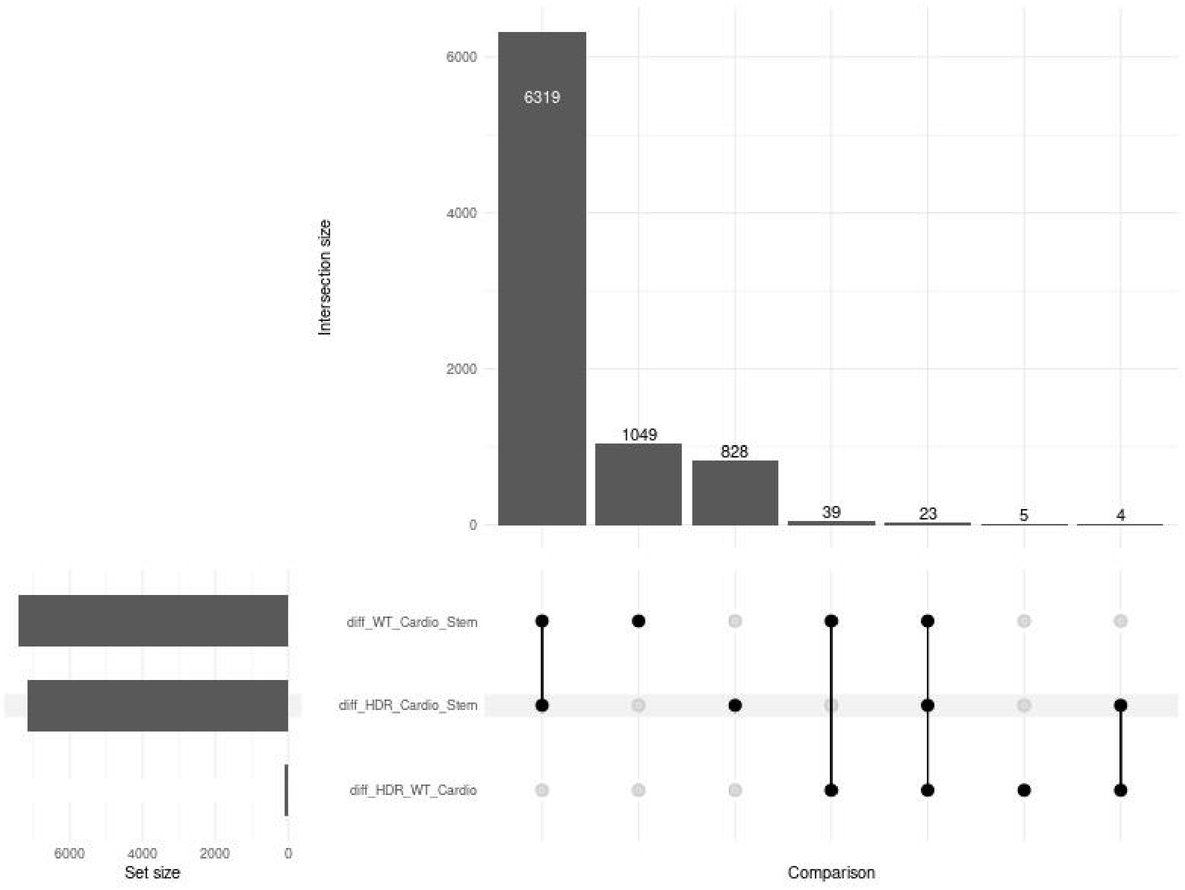

4.5 Gene set enrichment analysis (GSEA)

Here we use the clusterprofiler package[4] to interrogate our DE gene lists and perform GSEA.

4.5.1 Differentiation iPS to cardiomyocytes

Here we look for enriched terms in iPS to cardiomyocyte samples for both wild type and edited samples. The code below runs the enrichment analysis separately on WT / HDR then displays the results in a single figure.

First let's get the DE gene lists. Given the large number of genes I apply a more stringent logFC cutoff of 2 for the purpose of looking for enriched termsL

```
top <- topTreat(fit, coef= "diff_WT_Cardio_Stem", number=Inf)
geneList = top$logFC
names(geneList) = as.character(top$FeatureID)
geneList = sort(geneList, decreasing = TRUE)
```

```
WT <- names(geneList)[abs(geneList) > 2]

top <- topTreat(fit, coef= "diff_HDR_Cardio_Stem", number=Inf)
geneList = top$logFC
names(geneList) = as.character(top$FeatureID)
geneList = sort(geneList, decreasing = TRUE)
HDR <- names(geneList)[abs(geneList) > 2]
```

Append both lists and run the compare cluster analysis.

```
gc <- list(WT,HDR)

names(gc) = c("WT","HDR")

ck <- compareCluster(geneCluster = gc,
                     fun = enrichGO,
                     OrgDb='org.Hs.eg.db',
                     universe      = names(geneList),
                     ont           = "BP",
                     pAdjustMethod = "BH",
                     pvalueCutoff  = 0.01,
                     qvalueCutoff  = 0.05,
                     readable      = TRUE)



WT_and_HDR_diff <- dotplot(ck,showCategory = 25)

ggsave(file="Differentiation_WT_and_HDR_GOBP.pdf", plot=WT_and_HDR_diff , width=10,
   ↪ height=12)
```

Let's create a copy of the plot for the report

```
WT_and_HDR_diff
```

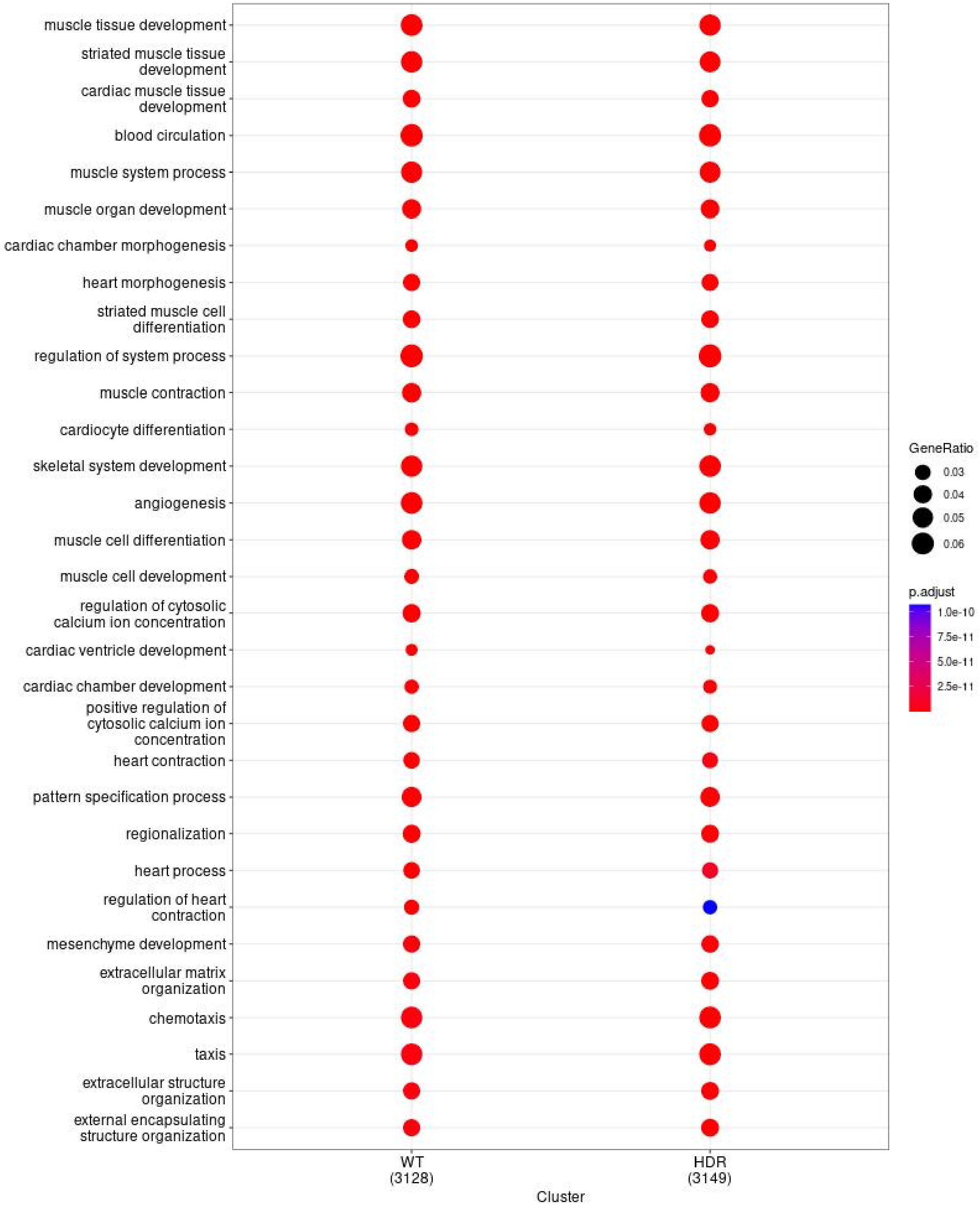
muscle tissue development
striated muscle tissue development
cardiac muscle tissue development
blood circulation
muscle system process
muscle organ development
cardiac chamber morphogenesis
heart morphogenesis
striated muscle cell differentiation
regulation of system process
muscle contraction
cardiocyte differentiation
skeletal system development
angiogenesis
muscle cell differentiation
muscle cell development
regulation of cytosolic calcium ion concentration
cardiac ventricle development
cardiac chamber development
positive regulation of cytosolic calcium ion concentration
heart contraction
pattern specification process
regionalization
heart process
regulation of heart contraction
mesenchyme development
extracellular matrix organization
chemotaxis
taxis
extracellular structure organization
external encapsulating structure organization
WT (3128)
HDR (3149)
Cluster
GeneRatio
0.03
0.04
0.05
0.06
p.adjust
1.0e-10
7.5e-11
5.0e-11
2.5e-11

4.5.2 Differences between WT and HDR

Here we look at differences between HDR and WT samples corrected by the baseline measurements at time-point.

Since there are no differentially expressed genes we will run a DisGeNET Gene Set Enrichment Analysis.

```
top <- topTreat(fit, coef= "diff_HDR_WT_Cardio_Stem", number=Inf)

geneList = top$logFC
names(geneList) = as.character(top$FeatureID)
geneList = sort(geneList, decreasing = TRUE)
```

Run the gsea analysis

```
dgn <- gseDGN(geneList,
              pvalueCutoff  = 0.01,
              pAdjustMethod = "BH",
              nPermSimple = 100000,
              by = "fgsea",
              seed = 42,
              eps = 0,
              ## universe      = names(geneList),
              verbose       = TRUE)
dgn <- setReadable(dgn, 'org.Hs.eg.db')
dgn <- pairwise_termsim(dgn)
```

Create overview plots :

```

p1 <- treeplot(dgn, hclust_method = "average", nWords = 2, hilight = FALSE,nCluster =
   ↪ 10,offset=1,hexpand = 0.5,offset_tiplab = 3 ) +
      theme(legend.position = "top")
p2 <- cnetplot(dgn, foldChange=geneList)
p3 <- emapplot(dgn)
## aplot::plot_list(DEgenesUpset, p1,p2,p3, tag_levels='A')


first_column = cowplot::plot_grid(DEgenesUpset, p3,labels = c('A','C'), ncol = 1)
second_column = cowplot::plot_grid(p1, labels = c('B'));
g <- cowplot::plot_grid(first_column, second_column, ncol = 2)

ggsave(file="UpSetDiffDiff.pdf", plot=g , width=18, height=12)
```

```
ggsave(file="DiffDiffGeneNetwork.pdf", plot=p2 , width=18, height=18)
```

For the report:

```
p1
```

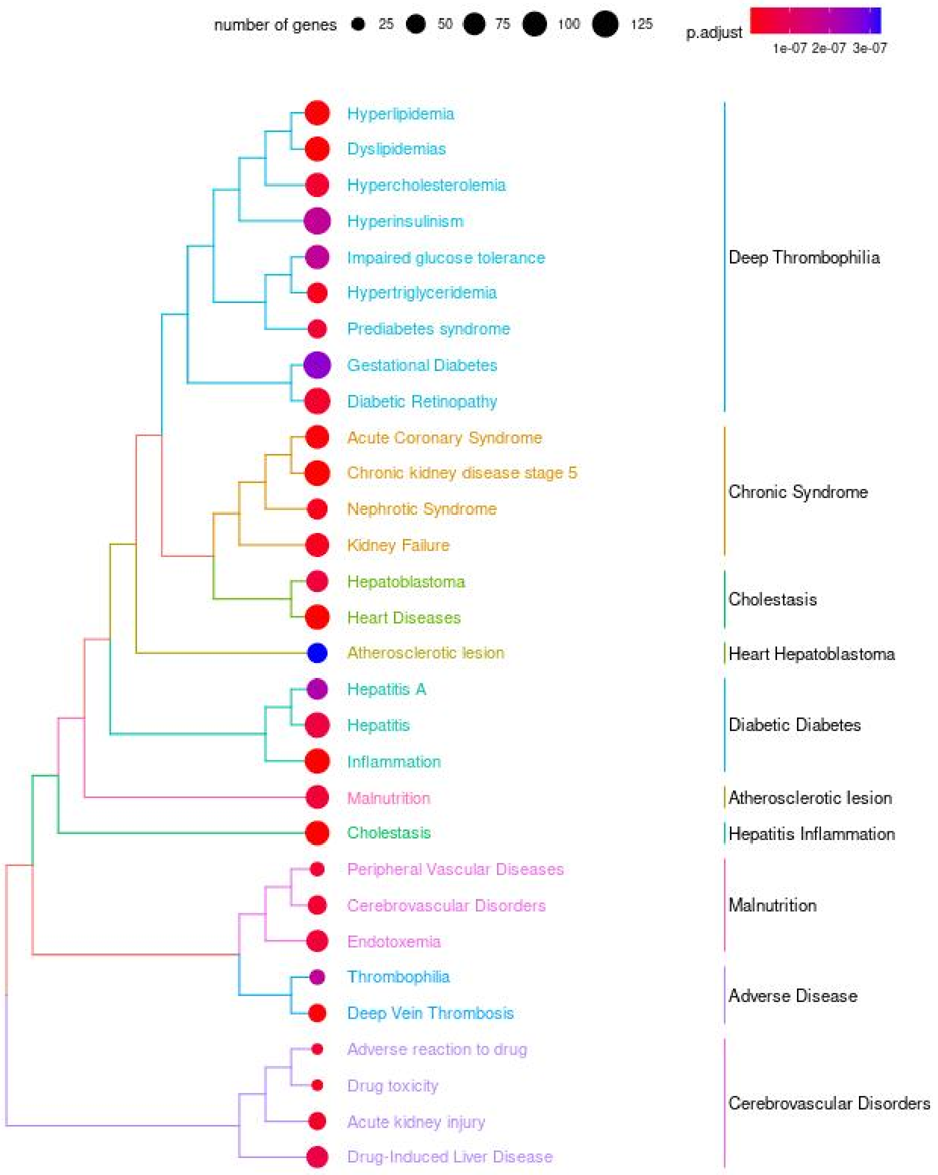


```
p2
```

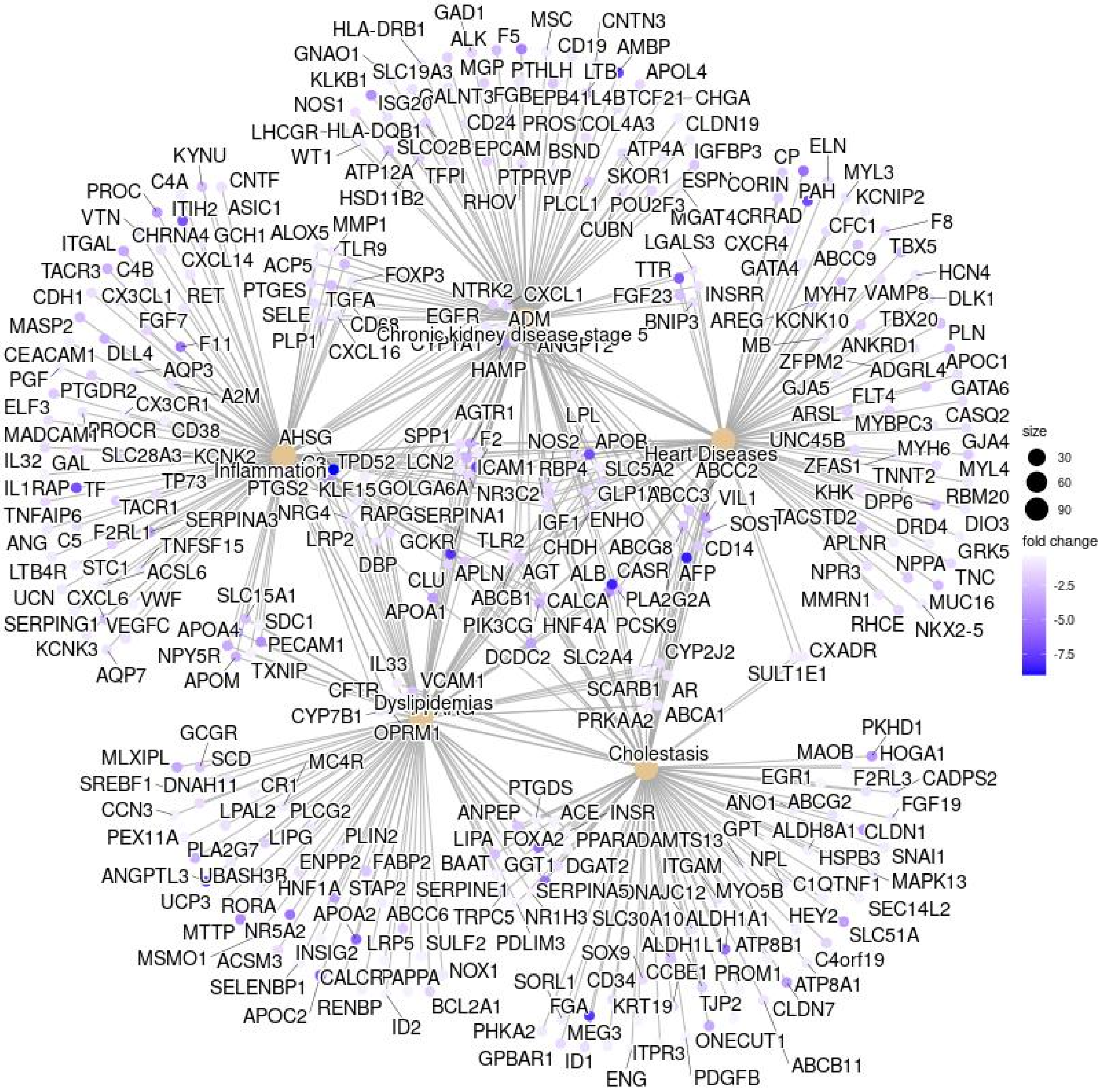
Chronic kidney disease stage 5
Inflammation
Heart Diseases
Dyslipidemias
Cholestasis
size
30
60
90
fold change
-2.5
-5.0
-7.5
GAD1
HLA-DRB1
ALK
F5
MSC
CNTN3
CD19
AMBP
GNAO1
SLC19A3
MGP
PTHLH
LTB
APOL4
KLKB1
NOS1
ISG20
GALNT3
FGB
EPB41L4B
TCF21
CHGA
LHCGR
HLA-DQB1
CD24
PROS
COL4A3
CLDN19
WT1
SLCO2B
EPCAM
BSND
ATP4A
IGFBP3
CP
ELN
KYNU
ATP12A
TFPI
PTPRVP
SKOR1
ESPN
CORIN
PAH
MYL3
PROC
C4A
CNTF
HSD11B2
RHOV
PLCL1
POU2F3
MGAT4C
RRAD
KCNIP2
VTN
ITIH2
ASIC1
MMP1
CUBN
CFC1
F8
ITGAL
CHRNA4
GCH1
ALOX5
TLR9
LGALS3
CXCR4
TBX5
TACR3
C4B
CXCL14
ACP5
FOXP3
TTR
GATA4
ABCC9
HCN4
CDH1
CX3CL1
RET
PTGES
NTRK2
CXCL1
FGF23
INSRR
MYH7
VAMP8
DLK1
MASP2
FGF7
SELE
TGFA
EGFR
ADM
BNIP3
AREG
KCNK10
TBX20
PLN
F11
PLP1
CD68
CXCL16
ANGPT2
MB
ANKRD1
CEACAM1
DLL4
AQP3
HAMP
ZFPM2
ADGRL4
APOC1
PGF
A2M
GJA5
GATA6
ELF3
PTGDR2
CX3CR1
AGTR1
LPL
FLT4
ARSL
CASQ2
MADCAM1
PROCR
CD38
AHSG
SPP1
F2
NOS2
APOB
MYBPC3
UNC45B
GJA4
IL32
GAL
SLC28A3
KCNK2
TPD52
LCN2
ICAM1
RBP4
SLC5A2
ABCC2
MYH6
ZFAS1
TNNT2
MYL4
IL1RAP
TF
TP73
PTGS2
KLF15
GOLGA6A
NR3C2
GLP1R
ABCC3
VIL1
KHK
DPP6
RBM20
TNFAIP6
TACR1
SERPINA3
NRG4
RAPGEF
SERPINA1
IGF1
ENHO
SOST
TACSTD2
DRD4
DIO3
ANG
C5
F2RL1
TNFSF15
LRP2
GCKR
TLR2
CHDH
ABCG8
CD14
APLNR
GRK5
LTB4R
STC1
ACSL6
DBP
CLU
APLN
AGT
ALB
CASR
AFP
NPR3
NPPA
TNC
UCN
CXCL6
VWF
SLC15A1
ABCB1
CALCA
PLA2G2A
MMRN1
MUC16
SERPING1
VEGFC
APOA4
SDC1
APOA1
PIK3CG
HNF4A
PCSK9
RHCE
NKX2-5
KCNK3
NPY5R
PECAM1
DCDC2
SLC2A4
CYP2J2
CXADR
AQP7
APOM
TXNIP
IL33
VCAM1
SULT1E1
CFTR
SCARB1
AR
CYP7B1
PRKAA2
ABCA1
OPRM1
PKHD1
GCGR
MAOB
HOGA1
MLXIPL
SCD
MC4R
EGR1
F2RL3
CADPS2
SREBF1
DNAH11
CR1
PTGDS
ANO1
ABCG2
FGF19
CCN3
LPAL2
PLCG2
ANPEP
ACE
INSR
GPT
ALDH8A1
CLDN1
PEX11A
LIPG
PLIN2
LIPA
FOXA2
PPARA
ADAMTS13
HSPB3
SNAI1
PLA2G7
ANGPTL3
UBASH3B
ENPP2
FABP2
BAAT
GGT1
DGAT2
ITGAM
NPL
MAPK13
UCP3
HNF1A
STAP2
SERPINE1
SERPINA5
DNAJC12
MYO5B
C1QTNF1
RORA
APOA2
ABCC6
TRPC5
NR1H3
SLC30A10
ALDH1A1
HEY2
SEC14L2
MTTP
NR5A2
LRP5
SULF2
PDLIM3
SOX9
ALDH1L1
ATP8B1
SLC51A
MSMO1
ACSM3
INSIG2
C4orf19
SELENBP1
CALCR
PAPPA
NOX1
SORL1
CD34
CCBE1
PROM1
ATP8A1
APOC2
RENBP
BCL2A1
FGA
KRT19
TJP2
CLDN7
ID2
PHKA2
MEG3
ONECUT1
GPBAR1
ID1
ITPR3
ABCB11
ENG
PDGFB

p3

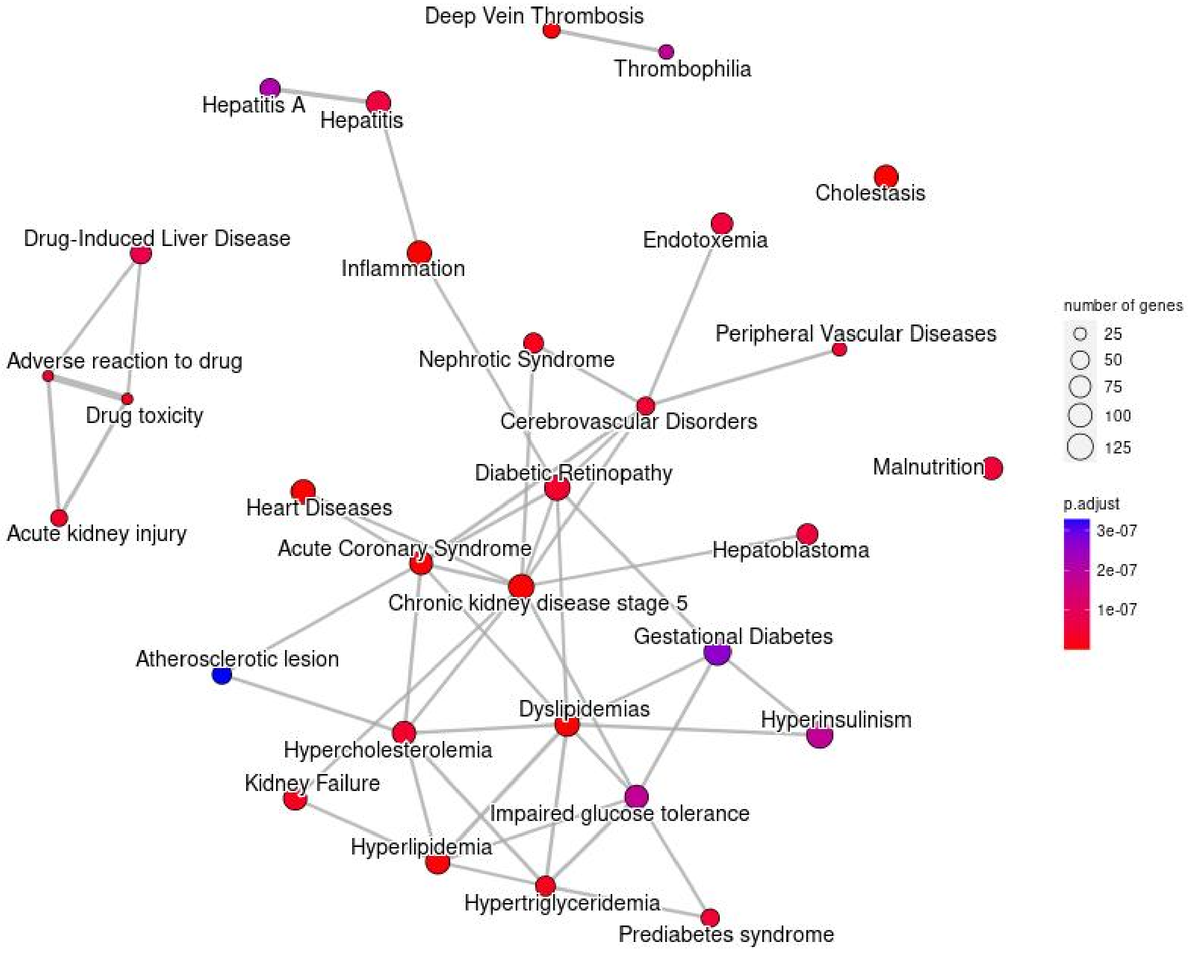

4.6 DONE Enrichment analysis

- Produce the index.html report
- discover terms relevant to patient phenotype

  GATA motif found in the ips -> Cardio / wt vs HDR comparison (!)

5 Setup / libraries

```
libraries <- c(
 "clusterProfiler",
  "org.Hs.eg.db",
  "BiocParallel",
  "tidyverse",
  "RColorBrewer",
  "limma",
  "edgeR",
  "EGSEA",
  "tximport",
  "DRIMSeq",
  "stageR",
  "GGally",
  "ggcorrplot",
  "ggdendro",
  "ggpubr",
  "ggbeeswarm",
  "xlsx",
  "ComplexUpset",
  "DOSE",
  "enrichplot"
)

lapply(libraries, FUN = function(X) {
  do.call("library", list(X))
})
setwd("../scratch")
```

```

DE_result_plot <- function (fit,dge,meta, contrast)
{
  cat( paste(contrast,"DE results\n"))
  top <- topTreat(fit,lfc=0.5,   coef= contrast, number=Inf)
  padj_cutoff <- 0.05
  top  <- dplyr::filter(top, adj.P.Val < padj_cutoff) %>% dplyr::arrange(adj.P.Val)%>%
    top_n(100)
  message(nrow(top))
```

```
  if(nrow(top) > 0){

    filename <- paste0("DEplots_",contrast,".pdf")
    top_gene_boxplot(dge ,meta, top,filename)
  }
}

top_gene_boxplot <- function(dge ,meta, top,filename)
{
  cpm <- cpm(dge)
  ## dds <- estimateSizeFactors(dds)
  colnames(cpm) <- meta$Sequencing_ID
  ## normalized_counts <- counts(dds, normalized = TRUE)
  colnames(top)[1] = "gene"
  top20_sig_norm <- data.frame(cpm) %>%
    rownames_to_column(var = "gene") %>%
    dplyr::filter(gene %in% rownames(top))

  tmp <- top %>% select(gene , Symbols)

  top20_sig_norm <- left_join(top20_sig_norm, tmp, by= "gene")
  top20_sig_norm <- top20_sig_norm %>% relocate(Symbols)
  top20_sig_norm <- top20_sig_norm %>% select(-gene)
  gathered_top20_sig <- top20_sig_norm %>%  gather(colnames(top20_sig_norm)[2:length(
   ↪ colnames(top20_sig_norm))], key = "samplename", value = "normalized_counts")

  gathered_top20_sig$samplename <- gsub("[.]","-",gathered_top20_sig$samplename)
  message(dim(top20_sig_norm))
  gathered_top20_sig <- inner_join(meta[, c("Sequencing_ID","Group", "Pheno" )],
   ↪ gathered_top20_sig, by = c("Sequencing_ID" = "samplename"))

  top$gene <- rownames(top)
  gathered_top20_sig <- inner_join(top[, c("Symbols","adj.P.Val" )], gathered_top20_
   ↪ sig, by = "Symbols")
  tmp <- tibble(Symbols =  gathered_top20_sig$Symbols, adj.P.Value =  formatC(gathered
   ↪ _top20_sig$adj.P.Val, format = "e", digits = 2))
  gathered_top20_sig$Symbols <-  paste0(tmp$Symbols ," (padj ", tmp$adj.P.Value,")")

  g <- ggplot(gathered_top20_sig) +
```

```
  geom_boxplot(aes(x = Pheno,
                   y = normalized_counts,
                   color = Group)) +
  ## geom_point(aes(x = Timepoint,
  ##                  y = normalized_counts,
  ##                  color = PFS_6M),
  ##              position=position_jitter(w=0.1,h=0)) +
  facet_wrap(~Symbols, nrow=5 , scales = "free") +
  scale_y_log10() +
  xlab("Genes") +
  ylab("log10 Normalized Counts") +
  ggtitle("Top DE Genes") +
  theme_bw() +
  theme(axis.text.x = element_text(angle = 45, hjust = 1)) +
  theme(plot.title = element_text(hjust = 0.5))
 ggsave(filename,g, width=40,height=30)
}
```

6 References (1 page)

Publications from the Research team are highlighted in bold.

1. Halchenko, Y. O., Hanke, M., Poldrack, B., Meyer, K., Solanky, D. S., Alteva, G., Gors, J., MacFarlane, D., Olaf Häusler, C., Olson, T., Waite, A., De La Vega, A., Sochat, V., Keshavan, A., Ma, F., Christian, H., Poelen, J., Skytén, K., Visconti di Oleggio Castello, M., Hardcastle, N., Stoeter, T., C Lau, V. & Markiewicz, C. J. *datalad/datalad 0.12.0rc6* version 0.12.0rc6. Oct. 2019. https://doi.org/10.5281/zenodo.3512712 (page 2).
2. Soneson, C., Love, M. I. & Robinson, M. D. Differential Analyses for Rna-Seq: Transcript-Level Estimates Improve Gene-Level Inferences. *F1000Research* **4** (1521 2015) (page 9).
3. Law, C. W., Alhamdoosh, M., Su, S., Dong, X., Tian, L., Smyth, G. K. & Ritchie, M. E. Rna-Seq Analysis Is Easy As 1-2-3 With Limma, Glimma and Edger. *F1000Research* **5** (2016) (page 10).
4. Wu, T., Hu, E., Xu, S., Chen, M., Guo, P., Dai, Z., Feng, T., Zhou, L., Tang, W., Zhan, L., *et al.* Clusterprofiler 4.0: a Universal Enrichment Tool for Interpreting Omics Data. *The Innovation* **2**, 100141 (2021) (page 22).

7 Appendix

7.1 Python virtual environment

Convenience script:

```bash
#!/usr/bin/env bash

DATALAD_ROOT=$(git rev-parse --show-toplevel)

DATALAD=$DATALAD_ROOT"/.datalad"
if [ ! -d "$DATALAD" ]; then
    printf "Directory: %s does not seem to be a datalad directory !\n" $DATALAD
    exit 1
fi

cd $DATALAD_ROOT

DIRECTORY=scratch/capercroo

mkdir -p scratch

printf "Detecting presence of existing environment %s \n" $CWD

if [ ! -d "$DIRECTORY" ]; then
    printf "The caper / croo environment was not found.\nSetting this up now\n"
    # Control will enter here if $DIRECTORY doesn't exist.
    virtualenv --python=python3 scratch/capercroo
    . scratch/capercroo/bin/activate
    test -z "$$VIRTUAL_ENV" && \
        echo "ERROR: must be executed in a virtual env (set VIRTUAL_ENV to fake one)"
    ↪ && \
        exit 1 || true
        echo "# Ensure dependency installation"
        python -m pip install -r ./pipeline_config/requirements.txt
    deactivate
fi

printf "\n\nTesting  caper / croo environment\n"
```

```
. scratch/capercroo/bin/activate
printf "Found at %s \n" $VIRTUAL_ENV

CAPER_VERSION=$(caper --version)
CROO_VERSION=$(croo  --version)

printf "   CAPER version %s \n" $CAPER_VERSION
printf "   CROO  version %s \n" $CROO_VERSION

printf "\n"

printf "To activate type:\n\n   . scratch/capercroo/bin/activate\n\n"
```

List of required software (from above: `requirements.txt`):

```
argcomplete==1.12.3
autouri==0.2.6
awscli==1.22.12
boto3==1.20.12
botocore==1.23.12
bullet==2.2.0
cachetools==4.2.4
caper==2.1.1
certifi==2021.10.8
cffi==1.15.0
charset-normalizer==2.0.7
colorama==0.4.3
coloredlogs==15.0.1
croo==0.6.0
cryptography==36.0.0
cycler==0.11.0
dateparser==1.1.0
docker==5.0.3
docutils==0.15.2
filelock==3.4.0
fonttools==4.28.2
google-api-core==2.2.2
google-auth==2.3.3
google-cloud-core==2.2.1
```

```
google-cloud-storage==1.43.0
google-crc32c==1.3.0
google-resumable-media==2.1.0
googleapis-common-protos==1.53.0
graphviz==0.18.2
humanfriendly==10.0
idna==3.3
importlib-metadata==4.8.2
jmespath==0.10.0
joblib==1.1.0
kiwisolver==1.3.2
lark-parser==0.12.0
matplotlib==3.5.0
miniwdl==1.3.3
numpy==1.21.4
packaging==21.3
pandas==1.3.4
Pillow==8.4.0
protobuf==3.19.1
psutil==5.8.0
pyasn1==0.4.8
pyasn1-modules==0.2.8
pycparser==2.21
pygtail==0.11.1
pyhocon==0.3.58
pyOpenSSL==21.0.0
pyparsing==3.0.6
python-dateutil==2.8.2
python-json-logger==2.0.2
pytz==2021.3
pytz-deprecation-shim==0.1.0.post0
PyYAML==5.4.1
regex==2021.11.10
requests==2.26.0
rsa==4.7.2
s3transfer==0.5.0
scikit-learn==1.0.1
scipy==1.7.2
setuptools-scm==6.3.2
```

```
six==1.16.0
threadpoolctl==3.0.0
tomli==1.2.2
tzdata==2021.5
tzlocal==4.1
urllib3==1.26.7
websocket-client==1.2.1
xdg==5.1.1
zipp==3.6.0
```

7.2 RNAseq pipeline wdl file

```
version 1.0

import "../pipelines/umitools.wdl"
import "../pipelines/rnaseq-var.wdl" as variantWorkflow

struct RuntimeEnvironment {
  String docker
  String singularity
}


struct Readgroup {
    String id
    String lib_id
    File R1
    File? R2
}

struct Sample {
    String id
    Array[Readgroup] readgroups
    String? gender
}

struct SampleConfig {
    Array[Sample] samples
```

```
}


workflow rnaseqcmpl {
  meta {
    author: "Timo Lassmann - modified from the ENCODE-DCC pipeline written by Otto
    ↪ Jolanki"
    version: "1.2.4"

    croo_out_def: "https://storage.googleapis.com/encode-pipeline-output-definition/
    ↪ bulkrna.output_definition.json"
    description: "Kallisto based Bulk-RNA pipeline."
    RSEMreference: {
      description: 'NARROWPEAK file for pooled true replicate.',
      group: 'input_genomic_data',
      help: 'Define if you want to start pipeline from PEAK files. Define if you have
    ↪ multiple biological replicates. Pooled true replicate means analysis on pooled
    ↪ biological replicates.'
    }
  }

  input {
    String? gatk_path_override
    String gatk_path = select_first([gatk_path_override, "/gatk/gatk"])

    File referenceFasta
    File referenceFastaFai
    File referenceFastaDict
    File annotationsGTF
    File dbsnpVCF
    File dbsnpVCFIndex

    File RSEMreference
    File somalierSiteList

    Array[File] knownVcfs
    Array[File] knownVcfsIndices

    File sampleConfigFile
```

```
  String runname
  Boolean umiDeduplication
  Boolean variantCalling
  String bcPattern
  String? bcPattern2

  Int? kallisto_number_of_threads
  Int? kallisto_ramGB
  File kallisto_index
  Int? trim_begin = 0
  Int? trim_end = 0
  File genes_gtf
  File star_index
  String singularity = "rnaseq-pipe"
  String docker = "encodedcc/rna-seq-pipeline:1.2.4"
}

RuntimeEnvironment runtime_environment = {
  "docker": docker,
  "singularity": singularity
}
call InputConverter as convertSampleConfig {
  input:
  runtime_environment=runtime_environment,
  samplesheet = sampleConfigFile,
  outputFile = runname + "/samples.json"
}

SampleConfig sampleConfig = read_json(convertSampleConfig.json)

scatter (sample in sampleConfig.samples) {
  scatter (readgroup in sample.readgroups) {
    if (umiDeduplication == true){
      call umitools.Extract as umi_extract {
        input:
        sampleName = sample.id,
        read1=readgroup.R1,
        read2=readgroup.R2,
        read1Output=sample.id+"_RG_"+readgroup.id+"_umi_extracted_R1.fastq.gz",
```

```
      read2Output=sample.id+"_RG_"+readgroup.id+"_umi_extracted_R2.fastq.gz",
      bcPattern=bcPattern,
      bcPattern2=bcPattern2,
    }
  }
  #umi_extract.extractedRead1
  File tmpR1 = select_first([umi_extract.extractedRead1,readgroup.R1])
  File tmpR2 = select_first([umi_extract.extractedRead2,readgroup.R2])
}
if (umiDeduplication == true){
  call star_align as star_dedup {
    input:
    inputR1 = tmpR1,
    inputR2 = tmpR2,
    prefix = sample.id,
    star_index = star_index,
    num_threads = 16,
    memory = 48,
    runtime_environment=runtime_environment,
  }

  call umitools.Dedup as Dedup {
    input:
    sampleName = sample.id,
    inputBam = star_dedup.bam_file,
    outputBamPath = sample.id+"_pseudo_dedup.bam",
    statsPrefix=sample.id+"_dedup_stats",
    paired=true,
  }

  call bam_to_fastq {
    input:
    InBam = Dedup.deduppedBam,
    out_prefix = sample.id ,
    memory = 16,
    num_threads = 4,
    runtime_environment=runtime_environment,
  }
  Array[File] bamtofq1 = [bam_to_fastq.OutRead1]
```

```
    Array[File] bamtofq2 = [bam_to_fastq.OutRead2]
  }
  Array[File] a  = select_first([bamtofq1,tmpR1])
  Array[File] b  = select_first([bamtofq2,tmpR2])

  call star_align as star {
    input:
    inputR1 = a,
    inputR2 = b,
    prefix = sample.id,
    star_index = star_index,
    num_threads = 16,
    memory = 48,
    runtime_environment=runtime_environment,
    outSAMattrRGline = "ID:rg1 SM:"+sample.id+" PL:illumina",
  }

  call get_unmapped_fastq {
    input:
    bam_file=star.bam_file,
    prefix = sample.id,
    runtime_environment=runtime_environment,
  }

  call star_get_strandness {
    input:
    read_counts = star.read_counts,
    out_prefix = sample.id,
    runtime_environment=runtime_environment,
  }

  call bam_to_signals {
    input:
    input_bam=star.bam_file,
    referenceFastaFai=referenceFastaFai,
    strandedness=star_get_strandness.strandedness,
    bamroot=sample.id+"_genome",
    ncpus=1,
    ramGB=2,
```

```
    runtime_environment=runtime_environment,
  }

  call somalier_extract{
    input:
    bam_file=star.bam_file,
    bam_file_index=star.bam_index,
    referenceFasta=referenceFasta,
    sitelist=somalierSiteList,
    prefix = sample.id,
    memory = 4,
    num_threads = 1,
    runtime_environment=runtime_environment,
  }

  File somalierex = select_first([somalier_extract.somalier_extract])

  call rnaseqc2 {
    input:
    runtime_environment=runtime_environment,
    bam_file = star.bam_file,
    genes_gtf = genes_gtf,
    sample_id = sample.id,
    strandedness = star_get_strandness.libtype_rnaseqc,
  }


  File tpm_gcts = select_first([rnaseqc2.gene_tpm])
  File count_gcts = select_first([rnaseqc2.gene_counts])
  File exon_count_gcts = select_first([rnaseqc2.exon_counts])
  File metrics_tsvs = select_first([rnaseqc2.metrics])

  call rsem {
    input:
    runtime_environment=runtime_environment,
    transcriptome_bam=star.transcriptome_bam,
    prefix=sample.id,
    rsem_reference=RSEMreference,
    num_threads=4,
```

```
    memory=8,
  }
  File rsem_genes = select_first([rsem.genes])
  File rsem_transcripts = select_first([rsem.isoforms])


  if(variantCalling){
    call variantWorkflow.rnaseqvar{
      input:
      bam=star.bam_file,
      bamIndex=star.bam_index,
      SampleName=sample.id,
      referenceFasta=referenceFasta,
      referenceFastaFai=referenceFastaFai,
      referenceFastaDict=referenceFastaDict,
      annotationsGTF=annotationsGTF,
      dbsnpVCF=dbsnpVCF,
      dbsnpVCFIndex=dbsnpVCFIndex,
      knownVcfs=knownVcfs,
      knownVcfsIndices=knownVcfsIndices,
    }
  }

  call kallisto_fast_pe as kallisto {
    input:
    runtime_environment=runtime_environment,
    fastqs_R1=a,
    fastqs_R2=b,
    strandedness = star_get_strandness.libtype_kallisto,
    kallisto_index=select_first([kallisto_index]),
    ncpus=16,
    ramGB=16,
    trim_begin=trim_begin,
    trim_end=trim_end,
    out_prefix=sample.id,
  }

}
```

```
  call rsem_aggregate_results {
    input:
    prefix=runname,
    memory=16,
    num_threads=1,
    rsem_isoforms=rsem_transcripts,
    rsem_genes=rsem_genes,
    runtime_environment=runtime_environment,
  }

  call rnaseqc2_aggregate {
    input:
    memory=16,
    num_threads=1,
    tpm_gcts= tpm_gcts,
    count_gcts=count_gcts,
    exon_count_gcts=exon_count_gcts,
    metrics_tsvs=metrics_tsvs,
    prefix=runname,
    runtime_environment=runtime_environment,
  }

  call somalier_relate{
    input:
    somalier=somalierex,
    prefix=runname,
    memory = 4,
    num_threads = 1,
    runtime_environment=runtime_environment,
  }

}

task somalier_extract {
  input {
    File bam_file
    File bam_file_index
    File referenceFasta
    File sitelist
```

```
    String prefix
    Int memory = 4
    Int num_threads = 1
    RuntimeEnvironment runtime_environment
  }

  command {
    somalier extract --sample-prefix ~{prefix}  -d extracted/ --sites ~{sitelist} -f ~
    ↪ {referenceFasta} ~{bam_file}
  }
  output {
    File somalier_extract = "extracted/"+ prefix+".somalier"
  }
  runtime {
    singularity: runtime_environment.singularity
    memory: "${memory}GB"
    cpu: "${num_threads}"
  }
  meta {
    author: "Timo Lassmann"
  }
}

task somalier_relate {
  input {
    Array[File] somalier
    Int memory = 4
    Int num_threads = 1
    String prefix
    RuntimeEnvironment runtime_environment
  }

  String expandedPrefix = prefix+"_somalier"
  command {
    somalier relate --min-ab 0.2 --output-prefix ~{expandedPrefix} ~{sep=" " somalier}
  }
  output {
     File somalierSamplesTSV = prefix+"_somalier.samples.tsv"
     File somalierPairsTSV = prefix+"_somalier.pairs.tsv"
```

```
    File somalierHTML = prefix+"_somalier.html"
  }

  runtime {
    singularity: runtime_environment.singularity
    memory: "${memory}GB"
    cpu: "${num_threads}"
  }

  meta {
    author: "Timo Lassmann"
  }

}

task get_unmapped_fastq {
  input{
    File bam_file
    String prefix
    Int memory = 4
    Int num_threads = 1
    RuntimeEnvironment runtime_environment
  }
  String outR1 = prefix+"_R1_unmapped.fastq"
  String outR2 = prefix+"_R2_unmapped.fastq"

  command {
    samtools view -f 12 244.Aligned.sortedByCoord.out.bam  | samtools sort -@ ~{num_
  ↪ threads} -u -n - -O | samtools fastq --threads ~{num_threads} -1 testR1.fastq.gz
  ↪  -2 testR2.fastq.gz -0 /dev/null -s /dev/null -n  -
    seqtk  sample -s 11 -2 testR1.fastq.gz 1000 > ~{outR1}
    seqtk  sample -s 11 -2 testR2.fastq.gz 1000 > ~{outR2}

  }
  output {
    File unmapped_R1 = "${prefix}_R1_unmapped.fastq"
    File unmapped_R2 = "${prefix}_R2_unmapped.fastq"
  }
```

```
  runtime {
    singularity: runtime_environment.singularity
    memory: "${memory}GB"
    cpu: "${num_threads}"
  }

  meta {
    author: "Timo Lassmann"
  }
}

task rnaseqc2 {
  input{
    File bam_file
    File genes_gtf
    String sample_id
    String? strandedness
    File? intervals_bed
    File? reference_fasta
    File? reference_fasta_index
    String? flags
    Int memory = "16"
    Int num_threads = 4
    RuntimeEnvironment runtime_environment
  }

  String strand_param =  if strandedness!="" then "--stranded " + strandedness  else "
   ↪ "

  command {
    set -euo pipefail
    echo $(date +"[%b %d %H:%M:%S] Running RNA-SeQC 2")
    touch ${sample_id}.fragmentSizes.txt
    touch ${sample_id}.gc_content.tsv
    rnaseqc ${genes_gtf} ${bam_file} . -s ${sample_id} ${"--bed " + intervals_bed} ${
   ↪ strand_param} ${"--fasta " + reference_fasta} -vv ${flags}
    echo "  * compressing outputs"
    gzip *.gct
    echo $(date +"[%b %d %H:%M:%S] done")
```

```
  }

  output {
    File gene_tpm = "${sample_id}.gene_tpm.gct.gz"
    File gene_counts = "${sample_id}.gene_reads.gct.gz"
    File exon_counts = "${sample_id}.exon_reads.gct.gz"
    File metrics = "${sample_id}.metrics.tsv"
    File gc_content = "${sample_id}.gc_content.tsv"
    File insertsize_distr = "${sample_id}.fragmentSizes.txt"
  }

  runtime {
    singularity: runtime_environment.singularity
    memory: "${memory}GB"
    cpu: "${num_threads}"
  }

  meta {
    author: "Francois Aguet"
  }
}

task bam_to_fastq {
  input{
    File InBam
    String out_prefix
    Int memory = 16
    Int num_threads = 4
    RuntimeEnvironment runtime_environment
  }

  String read1 = out_prefix+"_R1.fastq.gz"
  String read2 = out_prefix+"_R2.fastq.gz"
  String tmp1 = out_prefix+"_R1.fastq"
  String tmp2 = out_prefix+"_R2.fastq"

  String sortedBam = out_prefix+"_sorted.bam"
  command <<<
    samtools sort -@ ~{num_threads} -u -n -o ~{sortedBam}  ~{InBam}
```

```
    bedtools bamtofastq -i ~{sortedBam} -fq ~{tmp1} -fq2 ~{tmp2}
    gzip ~{tmp1}
    gzip ~{tmp2}
    rm ~{sortedBam}
  >>>

  output {
    File OutRead1 = read1
    File OutRead2 = read2
  }

  runtime {
    memory: "~{memory} GB"
    singularity: runtime_environment.singularity
    cpu: "${num_threads}"
  }
}

task star_get_strandness {
  input {
    File read_counts
    String out_prefix
    Int memory = 1
    Int num_threads = 1
    RuntimeEnvironment runtime_environment
  }

  String typefile = out_prefix+"_libtype.txt"
  String typefile_rnaseqc = out_prefix+"_rnaseqc.txt"
  String typefile_kallisto = out_prefix+"_kallisto.txt"
  String typefile_strandedness = out_prefix+"_strandedness.txt"
  String typefile_strandedness_direction = out_prefix+"_strandedness_direction.txt"

  command <<<

    zcat ~{read_counts} | grep -v "N_" | awk '{unst+=$2;forw+=$3;rev+=$4}END{print
   ↪ forw,rev,unst}' > libtypetesting.txt
    cat libtypetesting.txt | awk '{print $2/$1,$3/$1,$3/$2}' | awk '{if($1<0.3 && $
   ↪ 3>3)print "stranded";else if($1>3 && $2>3)print "reverse";else print "
```

```
    ↪ unstranded"}'  > ~{typefile_strandedness_direction}
    cat libtypetesting.txt | awk '{print $2/$1,$3/$1,$3/$2}' | awk '{if($1<0.3 && $
    ↪ 3>3)print "stranded";else if($1>3 && $2>3)print "stranded";else print "
    ↪ unstranded"}'  > ~{typefile_strandedness}
    cat libtypetesting.txt | awk '{print $2/$1,$3/$1,$3/$2}' | awk '{if($1<0.3 && $
    ↪ 3>3)print "fr";else if($1>3 && $2>3)print "rf";else print ""}'
    ↪       > ~{typefile_rnaseqc}
    cat libtypetesting.txt | awk '{print $2/$1,$3/$1,$3/$2}' | awk '{if($1<0.3 && $
    ↪ 3>3)print "--fr-stranded";else if($1>3 && $2>3)print "--rf-stranded";else print
    ↪  ""}' > ~{typefile_kallisto}
    cat libtypetesting.txt | awk '{print $2/$1,$3/$1,$3/$2}' | awk '{if($1<0.3 && $
    ↪ 3>3)print "stranded";else if($1>3 && $2>3)print "reverse";else print "
    ↪ unstranded"}'

  >>>
  output {
    String strandedness_direction = read_string(typefile_strandedness_direction)
    String strandedness           = read_string(typefile_strandedness)
    String libtype_rnaseqc        = read_string(typefile_rnaseqc)
    String libtype_kallisto       = read_string(typefile_kallisto)

  }
  runtime {
    memory: "~{memory} GB"
    singularity: runtime_environment.singularity
    cpu: "${num_threads}"
  }
}


task star_align {
  input {
    Array[File] inputR1
    Array[File] inputR2
    #File fastq1
    #File? fastq2
    String prefix
    File star_index
```

```
  # STAR options
  Int? outFilterMultimapNmax
  Int? alignSJoverhangMin
  Int? alignSJDBoverhangMin
  Int? outFilterMismatchNmax
  Float? outFilterMismatchNoverLmax
  Int? alignIntronMin
  Int? alignIntronMax
  Int? alignMatesGapMax
  String? outFilterType
  Float? outFilterScoreMinOverLread
  Float? outFilterMatchNminOverLread
  Int? limitSjdbInsertNsj
  String? outSAMstrandField
  String? outFilterIntronMotifs
  String? alignSoftClipAtReferenceEnds
  String? quantMode
  String? outSAMattrRGline
  String? outSAMattributes
  File? varVCFfile
  String? waspOutputMode
  Int? chimSegmentMin
  Int? chimJunctionOverhangMin
  String? chimOutType
  Int? chimMainSegmentMultNmax
  Int? chimOutJunctionFormat
  File? sjdbFileChrStartEnd

  Int memory = 48
  Int num_threads = 4
  RuntimeEnvironment runtime_environment
}
command {
  set -euo pipefail


  # extract index
  echo $(date +"[%b %d %H:%M:%S] Extracting STAR index")
  mkdir star_index
```

```
tar -xvvf ${star_index} -C star_index --strip-components=1

mkdir star_out
# placeholders for optional outputs
touch star_out/${prefix}.Aligned.toTranscriptome.out.bam
touch star_out/${prefix}.Chimeric.out.sorted.bam
touch star_out/${prefix}.Chimeric.out.sorted.bam.bai
touch star_out/${prefix}.ReadsPerGene.out.tab  # run_STAR.py will gzip

/opt/run_STAR.py \
star_index ~{sep=',' inputR1} ~{sep="," inputR2} ${prefix} \
--output_dir star_out \
${"--outFilterMultimapNmax " + outFilterMultimapNmax} \
${"--alignSJoverhangMin " + alignSJoverhangMin} \
${"--alignSJDBoverhangMin " + alignSJDBoverhangMin} \
${"--outFilterMismatchNmax " + outFilterMismatchNmax} \
${"--outFilterMismatchNoverLmax " + outFilterMismatchNoverLmax} \
${"--alignIntronMin " + alignIntronMin} \
${"--alignIntronMax " + alignIntronMax} \
${"--alignMatesGapMax " + alignMatesGapMax} \
${"--outFilterType " + outFilterType} \
${"--outFilterScoreMinOverLread " + outFilterScoreMinOverLread} \
${"--outFilterMatchNminOverLread " + outFilterMatchNminOverLread} \
${"--limitSjdbInsertNsj " + limitSjdbInsertNsj} \
${"--outSAMstrandField " + outSAMstrandField} \
${"--outFilterIntronMotifs " + outFilterIntronMotifs} \
${"--alignSoftClipAtReferenceEnds " + alignSoftClipAtReferenceEnds} \
${"--quantMode " + quantMode} \
${"--outSAMattrRGline " + outSAMattrRGline} \
${"--outSAMattributes " + outSAMattributes} \
${"--varVCFfile " + varVCFfile} \
${"--waspOutputMode " + waspOutputMode} \
${"--chimSegmentMin " + chimSegmentMin} \
${"--chimJunctionOverhangMin " + chimJunctionOverhangMin} \
${"--chimOutType " + chimOutType} \
${"--chimMainSegmentMultNmax " + chimMainSegmentMultNmax} \
${"--chimOutJunctionFormat " + chimOutJunctionFormat} \
${"--sjdbFileChrStartEnd " + sjdbFileChrStartEnd} \
--threads ${num_threads}
```

```
    rm -rf star_index
  }

  output {
    File bam_file = "star_out/${prefix}.Aligned.sortedByCoord.out.bam"
    File bam_index = "star_out/${prefix}.Aligned.sortedByCoord.out.bam.bai"
    File transcriptome_bam = "star_out/${prefix}.Aligned.toTranscriptome.out.bam"
    File chimeric_junctions = "star_out/${prefix}.Chimeric.out.junction.gz"
    File chimeric_bam_file = "star_out/${prefix}.Chimeric.out.sorted.bam"
    File chimeric_bam_index = "star_out/${prefix}.Chimeric.out.sorted.bam.bai"
    File read_counts = "star_out/${prefix}.ReadsPerGene.out.tab.gz"
    File junctions = "star_out/${prefix}.SJ.out.tab.gz"
    File junctions_pass1 = "star_out/${prefix}._STARpass1/${prefix}.SJ.pass1.out.tab.
    ↪ gz"
    Array[File] logs = ["star_out/${prefix}.Log.final.out", "star_out/${prefix}.Log.
    ↪ out", "star_out/${prefix}.Log.progress.out"]
  }

  runtime {
    memory: "~{memory} GB"
    singularity: runtime_environment.singularity
    cpu: "${num_threads}"
  }
  meta {
    author: "Francois Aguet"
  }
}



task kallisto_fast_pe {
  input {
    Array[File] fastqs_R1
    Array[File] fastqs_R2
    Int ncpus = 16
    String ramGB = "16G"
    String? disks
    String out_prefix = ""
```

```
    String out_dir = "kallisto_out"
    File kallisto_index
    Int? trim_begin = 0
    Int? trim_end = 0
    String strandedness
    RuntimeEnvironment runtime_environment
    #String singularity
  }
  command <<<
    mkdir ~{out_dir}
    # kallisto quant -i ~{kallisto_index} -o ~{out_dir}/test.un \
    # <(zcat  ~{fastqs_R1[0]} | head -n 200000) \
    # <(zcat  ~{fastqs_R2[0]} | head -n 200000)

    # kallisto quant --rf-stranded -i ~{kallisto_index} -o ~{out_dir}/test.rf \
    # <(zcat  ~{fastqs_R1[0]} | head -n 200000) \
    # <(zcat  ~{fastqs_R2[0]} | head -n 200000)

    # kallisto quant --fr-stranded -i ~{kallisto_index} -o ~{out_dir}/test.fr \
    # <(zcat  ~{fastqs_R1[0]} | head -n 200000) \
    # <(zcat  ~{fastqs_R2[0]} | head -n 200000)

    # paste ~{out_dir}/test.fr/abundance.tsv ~{out_dir}/test.rf/abundance.tsv ~{out_dir}/test.un/abundance.tsv | \
    # cut -f1,4,9,14  | \
    # awk 'BEGIN{sum1=0;sum2=0;sum3=0}{sum1+=$2;sum2+=$3;sum3+=$4}END{print sum1,sum2,sum3}' > ~{out_dir}/test.libtypetesting

    # strand=$(cat ~{out_dir}/test.libtypetesting | awk '{print $2/$1,$3/$1,$3/$2}' | awk '{if($1<0.3 && $3>3)print "--fr-stranded";else if($1>3 && $2>3)print "--rf-stranded";else print ""}')

    kallisto quant --bootstrap-samples 100 ~{strandedness} -t ~{ncpus} -i ~{kallisto_index} -o  ~{out_dir} \
    <(zcat ~{sep=' ' fastqs_R1} | seqtk trimfq -b ~{trim_begin}  -e ~{trim_end} -) \
    <(zcat ~{sep=' ' fastqs_R2} | seqtk trimfq -b ~{trim_begin}  -e ~{trim_end} -)

    mv ~{out_dir}"/abundance.tsv"        ~{out_dir}/~{out_prefix}"_abundance.tsv"
    mv ~{out_dir}"/abundance.h5"         ~{out_dir}/~{out_prefix}"_abundance.h5"
```

```
    mv ~{out_dir}"/run_info.json"        ~{out_dir}/~{out_prefix}"_run_info.json"

  >>>

  output {
    File quants = out_dir+"/"+out_prefix+"_abundance.tsv"
    File quants_hdf5 = out_dir+"/"+out_prefix+"_abundance.h5"
    File run_info = out_dir+"/"+out_prefix+"_run_info.json"
  }

  runtime {
    cpu: ncpus
    memory: "~{ramGB} GB"
    disks: disks
    singularity: runtime_environment.singularity
  }
}




task InputConverter {
  input {
    File samplesheet
    String outputFile = "samplesheet.json"
    # File checking only works when:
    # 1. Paths are absolute
    # 2. When containers have the directory with the files mounted.
    # Therefore this functionality does not work well with cromwell.
    Boolean skipFileCheck=true
    Boolean checkFileMd5sums=false
    Boolean old=false

    String memory = "128M"
    Int timeMinutes = 1
    RuntimeEnvironment runtime_environment
    #String singularity = ""
  }
```

```
  command <<<
    set -e
    mkdir -p "$(dirname ~{outputFile})"
    biowdl-input-converter \
    -o ~{outputFile} \
    ~{true="--skip-file-check" false="" skipFileCheck} \
    ~{true="--check-file-md5sums" false="" checkFileMd5sums} \
    ~{true="--old" false="" old} \
    ~{samplesheet}
  >>>

  output {
    File json = outputFile
  }

  runtime {
    memory: memory
    singularity: runtime_environment.singularity
  }

  parameter_meta {
    # inputs
    samplesheet: {description: "The samplesheet to be processed.", category: "required
   ↪ "}
    outputFile: {description: "The location the JSON representation of the samplesheet
   ↪  should be written to.", category: "advanced"}
    skipFileCheck: {description: "Whether or not the existance of the files mentioned
   ↪ in the samplesheet should be checked.", category: "advanced"}
    checkFileMd5sums: {description: "Whether or not the MD5 sums of the files
   ↪ mentioned in the samplesheet should be checked.", category: "advanced"}
    old: {description: "Whether or not the old samplesheet format should be used.",
   ↪ category: "advanced"}
    memory: {description: "The amount of memory needed for the job.", category: "
   ↪ advanced"}
    # outputs
    json: {description: "JSON file version of the input sample sheet."}
  }
}
```

```
task  bam_to_signals {
  input {
    File? null
    File input_bam
    File referenceFastaFai
    String strandedness
    String bamroot
    Int ncpus
    Int ramGB
    RuntimeEnvironment runtime_environment
  }

  command {
    cat ~{referenceFastaFai} | cut -f 1,2 > chr_size.tsv
    python3 /opt/bam_to_signals.py \
    --bamfile ~{input_bam} \
    --chrom_sizes chr_size.tsv \
    --strandedness ~{strandedness} \
    --bamroot ~{bamroot}
  }

  output {
    File? unique_unstranded = if (strandedness == "unstranded") then glob("*_genome_
  ↪ uniq.bw")[0] else null
    File? all_unstranded = if (strandedness == "unstranded") then glob("*_genome_all.
  ↪ bw")[0] else null
    File? unique_plus = if (strandedness == "stranded") then glob("*_genome_plusUniq.
  ↪ bw")[0] else null
    File? unique_minus = if (strandedness == "stranded") then glob("*_genome_minusUniq
  ↪ .bw")[0] else null
    File? all_plus = if (strandedness == "stranded") then glob("*_genome_plusAll.bw")
  ↪ [0] else null
    File? all_minus = if (strandedness == "stranded") then glob("*_genome_minusAll.bw"
  ↪ )[0] else null
    File python_log = "bam_to_signals.log"
  }

  runtime {
    cpu: ncpus
```

```
    memory: "~{ramGB} GB"
    singularity: runtime_environment.singularity
  }
}

task rsem {
  input {
    File transcriptome_bam
    File rsem_reference
    String prefix

    Int memory

    Int num_threads
    RuntimeEnvironment runtime_environment

    Int? max_frag_len
    String? estimate_rspd
    String? is_stranded
    String? paired_end
  }
  command {
    set -euo pipefail
    mkdir rsem_reference
    tar -xvvf ${rsem_reference} -C rsem_reference --strip-components=1

    /opt/run_RSEM.py \
    ${"--max_frag_len " + max_frag_len} \
    ${"--estimate_rspd " + estimate_rspd} \
    ${"--is_stranded " + is_stranded} \
    ${"--paired_end " + paired_end} \
    --threads ${num_threads} \
    rsem_reference ${transcriptome_bam} ${prefix}
    gzip *.results
  }

  output {
    File genes="${prefix}.rsem.genes.results.gz"
    File isoforms="${prefix}.rsem.isoforms.results.gz"
```

```
  }

  runtime {

    memory: "${memory}GB"
    cpu: "${num_threads}"
    singularity: runtime_environment.singularity
  }

  meta {
    author: "Francois Aguet"
  }
}

task rsem_aggregate_results {

  input {
    Array[File] rsem_isoforms
    Array[File] rsem_genes
    String prefix

    Int memory

    Int num_threads
    RuntimeEnvironment runtime_environment
  }
  command {
    echo $(date +"[%b %d %H:%M:%S] Combining transcript-level output")
    python3 /opt/aggregate_rsem_results.py ${write_lines(rsem_isoforms)} TPM IsoPct
   ↪ expected_count ${prefix}
    echo $(date +"[%b %d %H:%M:%S] Combining gene-level output")
    python3 /opt/aggregate_rsem_results.py ${write_lines(rsem_genes)} TPM expected_
   ↪ count ${prefix}
  }

  output {
    File transcripts_tpm="${prefix}.rsem_transcripts_tpm.txt.gz"
    File transcripts_isopct="${prefix}.rsem_transcripts_isopct.txt.gz"
    File transcripts_expected_count="${prefix}.rsem_transcripts_expected_count.txt.gz"
```

```
    File genes_tpm="${prefix}.rsem_genes_tpm.txt.gz"
    File genes_expected_count="${prefix}.rsem_genes_expected_count.txt.gz"
  }

  runtime {
    memory: "${memory}GB"
    cpu: "${num_threads}"
    singularity: runtime_environment.singularity
  }

  meta {
    author: "Francois Aguet"
  }
}

task rnaseqc2_aggregate {
  input {
    Array[File] tpm_gcts
    Array[File] count_gcts
    Array[File] exon_count_gcts
    Array[File] metrics_tsvs
    String prefix
    Array[File]? insertsize_hists
    String? flags

    Int memory

    Int num_threads
    RuntimeEnvironment runtime_environment
  }

  command {
    set -euo pipefail
    echo $(date +"[%b %d %H:%M:%S] Aggregating RNA-SeQC outputs")
    mkdir individual_outputs
    mv ${sep=' ' tpm_gcts} individual_outputs/
    mv ${sep=' ' count_gcts} individual_outputs/
    mv ${sep=' ' exon_count_gcts} individual_outputs/
    mv ${sep=' ' metrics_tsvs} individual_outputs/
```

```
    if [ -n '${sep=',' insertsize_hists}' ]; then
    mv ${sep=' ' insertsize_hists} individual_outputs/
    fi
    touch ${prefix}.insert_size_hists.txt.gz
    python3 -m rnaseqc aggregate \
    -o . \
    individual_outputs \
    ${prefix} \
    ${flags}
    echo $(date +"[%b %d %H:%M:%S] done")
  }

  output {
    File metrics="${prefix}.metrics.txt.gz"
    File insert_size_hists="${prefix}.insert_size_hists.txt.gz"
    File tpm_gct=glob("${prefix}.gene_tpm.*")[0]
    File count_gct=glob("${prefix}.gene_reads.*")[0]
    File exon_count_gct=glob("${prefix}.exon_reads.*")[0]
  }

  runtime {
    memory: "${memory}GB"
    cpu: "${num_threads}"
    singularity: runtime_environment.singularity
  }

  meta {
    author: "Francois Aguet"
  }
}
```

7.3 Default cromwell config & backend

Config:

```
backend=slurm

slurm-partition=main
```

```
slurm-account=

slurm-resource-param=-n 1 --ntasks-per-node=1 --cpus-per-task=${cpu} ${if defined(
    ↪ memory_mb) then "--mem=" else ""}${memory_mb}${if defined(memory_mb) then "M"
    ↪ else ""} ${if defined(time) then "--time=" else ""}${time*60} ${if defined(gpu)
    ↪  then "--gres=gpu:" else ""}${gpu}

backend-file=./pipeline_config/backend.conf

local-hash-strat=path+modtime

local-loc-dir=./scratch
local-out-dir=./scratch


cromwell=./pipeline_config/cromwell-71.jar
womtool=./pipeline_config/womtool-71.jar
```

Backend:

```
include required(classpath("application"))
backend {
  default = "slurm"
  providers {
    slurm {
      config {
        default-runtime-attributes {
          slurm_partition = "main"
        }
        filesystems {
          local {
            caching {
              duplication-strategy = [
                "soft-link"
                "hard-link"
                "copy"
              ]
              check-sibling-md5 = true
              hashing-strategy = "path+modtime"
```

```
            }
            localization = [
              "soft-link"
              "hard-link"
              "copy"
            ]
          }
        }
        concurrent-job-limit = 1000
        script-epilogue = "sleep 5"
        run-in-background = true
        runtime-attributes = """
## Caper custom attributes
# Environment choices = (docker, conda, singularity)
# If environment is not specified then prioritize docker > singularity > conda
# gpu is a plain string (to be able to specify gpu's name)
String? environment
String? conda
String? singularity
String? singularity_bindpath
String? gpu


Int cpu = 1
Int? time
Int? memory_mb


String? slurm_partition
String? slurm_account
String? slurm_extra_param
"""
        submit = """
cat << EOF > ${script}.caper
#!/bin/bash


if [ '${defined(environment)}' == 'true' ] && [ '${environment}' == 'singularity' ] ||
   ↪  \
```

```
   [ '${defined(environment)}' == 'false' ] && [ '${defined(singularity)}' == 'true' ]
   ↪  && [ ! -z '${singularity}' ]
then
    mkdir -p $HOME/.singularity/lock/
    flock --exclusive --timeout 600  $HOME/.singularity/lock/`echo -n '${singularity}'
   ↪  | md5sum | cut -d' ' -f1` singularity exec --containall  $(find $(git rev-
   ↪ parse --show-toplevel) -name ${singularity})/image echo 'Successfully pulled ${
   ↪ singularity}'


    singularity exec --cleanenv --home=`dirname ${cwd}` \
        --bind=${singularity_bindpath}, \
        ${if defined(gpu) then ' --nv' else ''} \
        $(find $(git rev-parse --show-toplevel) -name ${singularity})/image ${job_
   ↪ shell} ${script}

elif [ '${defined(environment)}' == 'true' ] && [ '${environment}' == 'conda' ] || \
     [ '${defined(environment)}' == 'false' ] && [ '${defined(conda)}' == 'true' ] &&
   ↪ [ ! -z '${conda}' ]
then
    conda run --name=${conda} ${job_shell} ${script}

else
    ${job_shell} ${script}
fi

EOF

for ITER in 1 2 3
do
    sbatch --export=ALL -J ${job_name}  -o ${out} -e ${err} \
        ${'-p ' + slurm_partition} ${'--account ' + slurm_account} \
        -n 1 --ntasks-per-node=1 --cpus-per-task=${cpu} ${if defined(memory_mb) then "
   ↪ --mem=" else ""}${memory_mb}${if defined(memory_mb) then "M" else ""} ${if
   ↪ defined(time) then "--time=" else ""}${time*60} ${if defined(gpu) then "--gres=
   ↪ gpu:" else ""}${gpu} \
        ${slurm_extra_param} \
        ${script}.caper && exit 0
    sleep 30
```

```
done
exit 1
"""
        submit-docker = null
        kill-docker = null
        root = "./scratch"
        check-alive = """
for ITER in 1 2 3
do
    CHK_ALIVE=$(squeue --noheader -j ${job_id} --format=%i | grep ${job_id})
    if [ -z "$CHK_ALIVE" ]
    then
        if [ "$ITER" == 3 ]
        then
            ${job_shell} -c 'exit 1'
        else
            sleep 30
        fi
    else
        echo $CHK_ALIVE
        break
    fi
done
"""
        kill = "scancel ${job_id}"
        job-id-regex = "Submitted batch job ([0-9]+).*"
      }
      actor-factory = "cromwell.backend.impl.sfs.config.
   ↪ ConfigBackendLifecycleActorFactory"
    }
    }
}
webservice {}
services {
  LoadController {
    class = "cromwell.services.loadcontroller.impl.LoadControllerServiceActor"
    config {
      control-frequency = "21474834 seconds"
    }
```

```
  }
}
system {
  job-rate-control {
    jobs = 1
    per = "2 seconds"
  }
  abort-jobs-on-terminate = true
  graceful-server-shutdown = true
  max-concurrent-workflows = 40
}
call-caching {
  invalidate-bad-cache-results = true
  enabled = true
}
akka {
  http {
    server {
      request-timeout = "60 seconds"
    }
  }
}
database {
  db {
    connectionTimeout = 30000
    numThreads = 1
  }
}
```
