# Supplementary material for: Gene editing and cardiac disease modelling for the interpretation of genetic variants of uncertain significance in congenital heart disease
Source: Stem Cell Res Ther. 2023 Dec 5;14:345. doi: 10.1186/s13287-023-03592-1 (PMC10696868; doi:10.1186/s13287-023-03592-1)
Supplement: Supplementary file 2 — Additional file 2: Figure S1 Human Phenotype Ontology mapping. A Petient HPO terms. B Human phenotype ontology tree indicating parent and daughter HPO terms. Figure S2 GATA4 p.Arg283Cys interacts with cardiac transcription factor NKX2.5. A and B Protein structure of GATA4 indicating carboxy zinc finger domain (C’ Znf), and site Arginine to Cysteine mutation. C Introduction of GATA4pArg383Cys mutation in HEK293T cells and deep amplicon sequencing to determine genetic variant introduction in genomic DNA. D GATA4_WT (WT/WT), and homozygous GATA4_VUS (HDR/HDR) HEK293 cell protein expression for GATA4, and bactin by western blot. E, Immunoprecipitation of NKX2.5 in HEK293 GATA4_WT (WT/WT) and GATA4_VUS (HDR/HDR) indicated complex formation with GATA4. bactin expression in lysate preparation by western blot. Figure S3 GATA4_WT and GATA4_HDR clonal genotypes. CRISPResso analysis of amplicon sequencing demonstrating WT and genetic variant allele expression. A Total reads per cell line amplicon. B Frequency of genetic variant and WT representation. C GATA4 cell clones were amplicon sequenced. FASTQ data file from CRISPResso analysis as indicated. D Sanger sequencing histograms of WT and genetic variant GATA4 clones as indicated. Solid blue arrow indicates patient C > T, p.Arg283Cys; dotted line indicates silent mutation G > C p.Leu281 = . Figure S4 A Off-target sites and primer sequences. B Representative sanger sequencing at off-target sites. * Reverse complement sequence. Figure S5 Global changes comparing, and contrasting, GATA4_WT and GATA4_HDR cells. A Principal component analysis indicating iPSCs and cardiomyocytes for GATA4_WT or GATA4_HDR cells. B Difference in differentiation gene network changes. C Additional cardiac terms in DisGenNet Enrichment for comparison of cardiac differentiation between GATA4_WT or GATA4_HDR. Figure S6 Calcium and adrenergic signalling changes in cardiac differentiation. Gene set enrichment analysis using KEGG pathways. A GATA4_WT iPSC to [file 13287_2023_3592_MOESM2_ESM.pdf]

Supplemental Figures

A

Patient HPO terms	
HP:0001407	Hepatic cysts
HP:0001943	Hypoglycemia
HP:0001263	Global developmental delay
HP:0002019	Constipation
HP:0002194	Delayed gross motor development
HP:0000527	Long eyelashes
HP:0002028	Chronic diarrhea
HP:0001252	Muscular hypotonia
HP:0000316	Hypertelorism
HP:0006695	Atrioventricular canal defect
HP:0001792	Small nail
HP:0000750	Delayed speech and language development
HP:0001633	Abnormality of the mitral valve
HP:0000324	Facial asymmetry

B

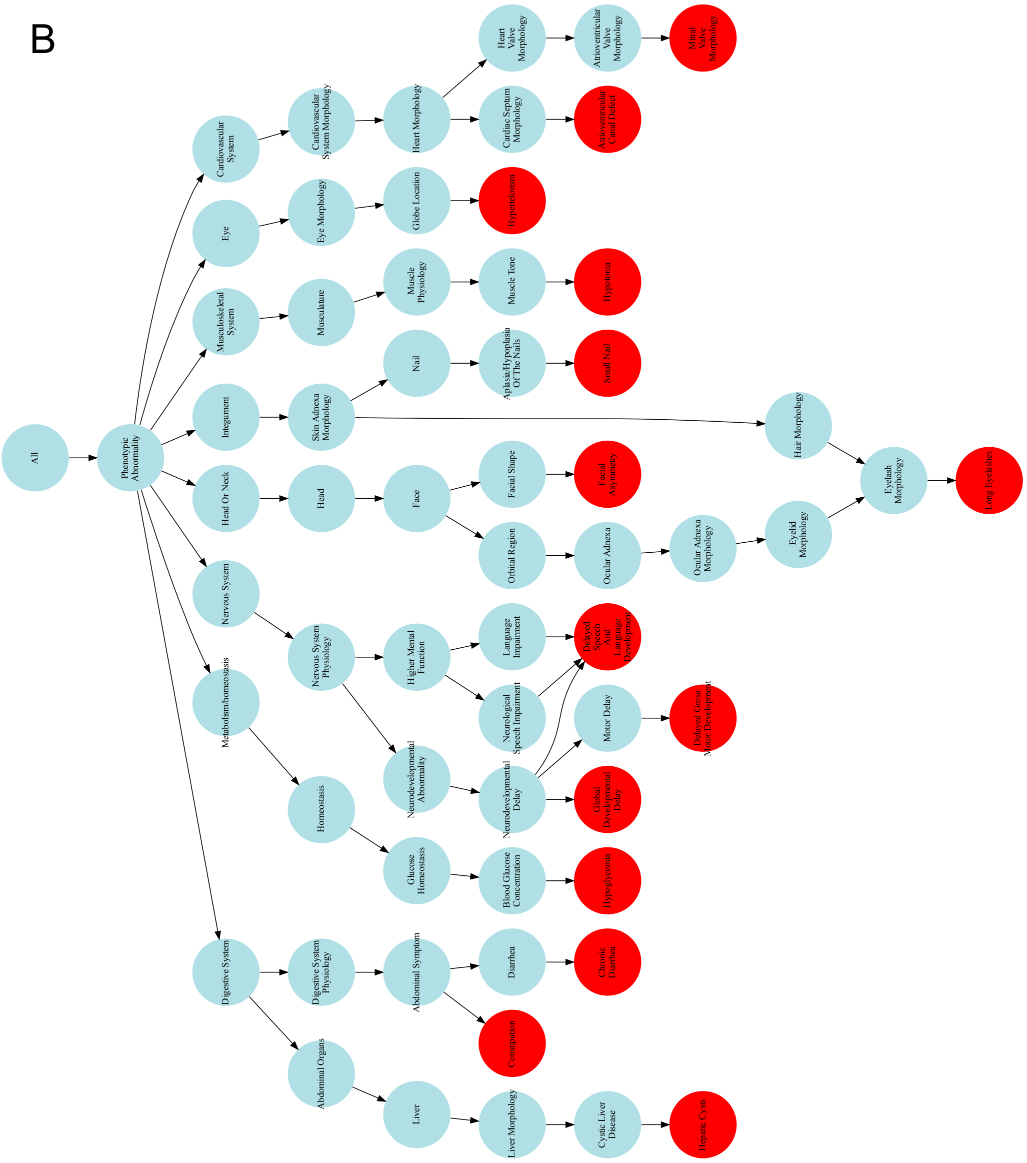

Figure S1. Human Phenotype Ontology mapping. A, Petient HPO terms. B, Human phenotype ontology tree indicating parent and daughter HPO terms.

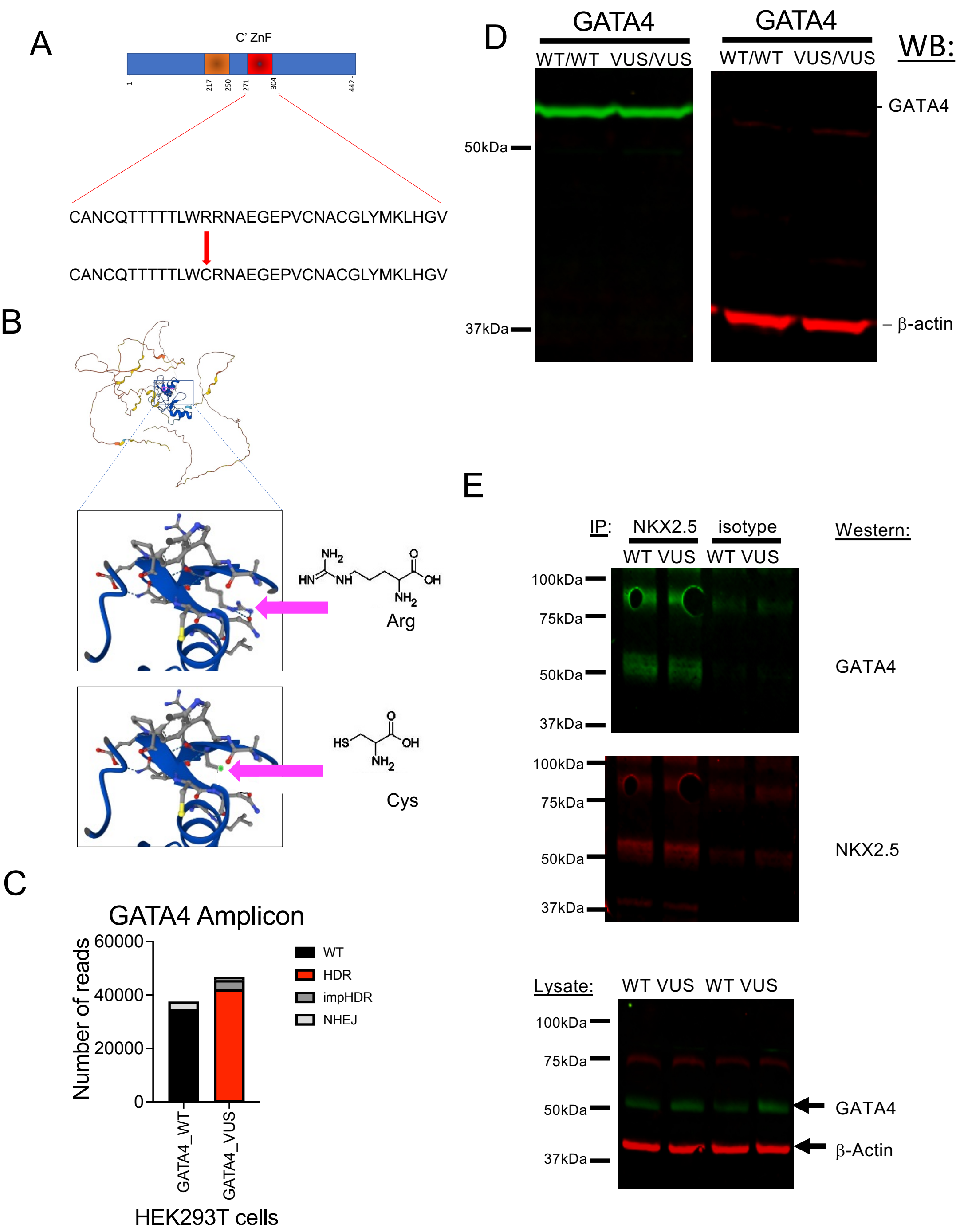

Figure S2. GATA4 p.Arg283Cys interacts with cardiac transcription factor NKX2.5. A and B, Protein structure of GATA4 indicating carboxy zinc finger domain (C' Znf), and site Arginine to Cysteine mutation. C, Introduction of GATA4pArg383Cys mutation in HEK293T cells and deep amplicon sequencing to determine genetic variant introduction in genomic DNA. D, *GATA4*_WT (WT/WT), and homozygous *GATA4*_VUS (HDR/HDR) HEK293 cell protein expression for GATA4, and b-actin by western blot. E, Immunoprecipitation of NKX2.5 in HEK293 *GATA4*_WT (WT/WT) and *GATA4*_VUS (HDR/HDR) indicated complex formation with GATA4. b-actin expression in lysate preparation by western blot.

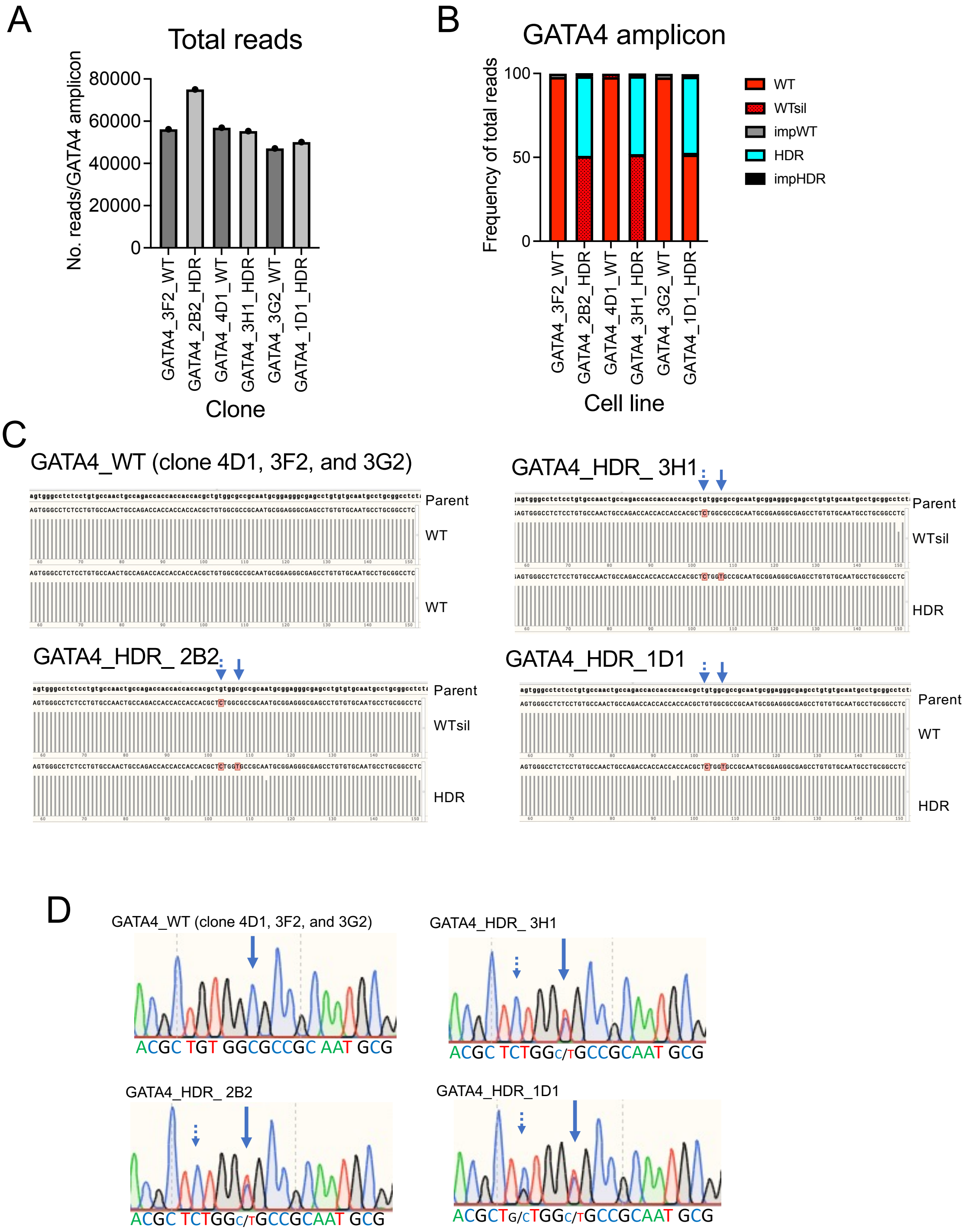

Figure S3. GATA4_WT and GATA4_HDR clonal genotypes. Crispresso analysis of amplicon sequencing demonstrating WT and genetic variant allele expression. (A) Total reads per cell line amplicon. (B) frequency of genetic variant and WT representation. (C) GATA4 cell clones were amplicon sequenced. Fastq data file from Crispresso analysis as indicated. (D) Sanger sequencing histograms of WT and genetic variant GATA4 clones as indicated. Solid blue arrow indicates patient C>T, p.Arg283Cys; dotted line indicates silent mutation G>C p.Leu281=.

A

	Sequence	PAM	Score	#MM	Gene	Locus	F primer	R primer	product size
	AGACCACCACCACCACGCTG	TGG	N/A		GATA4	chr8:+11750150			
OT1	AAACCACTACCACCACACTG	AGG	5	3		chr11:+93522840	GCCAAGTCTCCGCCATTGTA	ACCAGCAAAGTGACCTGCAA	433bp
OT2	GCACCACTACCACCACACTG	CAG	5	4		chr10:-33126635	CAGGCCGCATGAGGAATAGA	ATGCCGTCTTGCTTTGCTTG	419bp
OT3	AGATCAC-ACCACCACACTG	CAG	10	3		chr13:-70554342	GCATGTACTATGGTCAGGCA	TCAATAACACTGCATTGTGGCA	401bp
OT4	AGGCCCACACCACCACACTG	GGG	17	4		chr8:+49479239	ACCCAACCCTTGGTAAACAGT	TGCTGACCCAGACATTTCTTCA	481bp
OT5	AAAACAACACCACCACACTG	GGG	19	4		chr15:+64456050	AGTCTGGCCGTCCTTAGTGA	AGAATCGGGTGGGTTTTCCC	400bp
OT6	CCACCACCACCCCCACACTG	CAG	22	4		chr9:-35846101	TGGGTGGAGATGGAGGATGT	CACCGCTCACCGAGGC	469bp

B

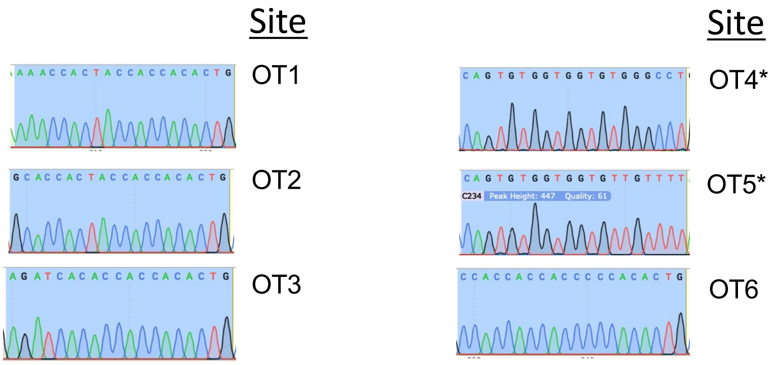

Figure S4. A, Off-target sites and primer sequences. B, Representative sanger sequencing at off target sites. * Reverse complement sequence.

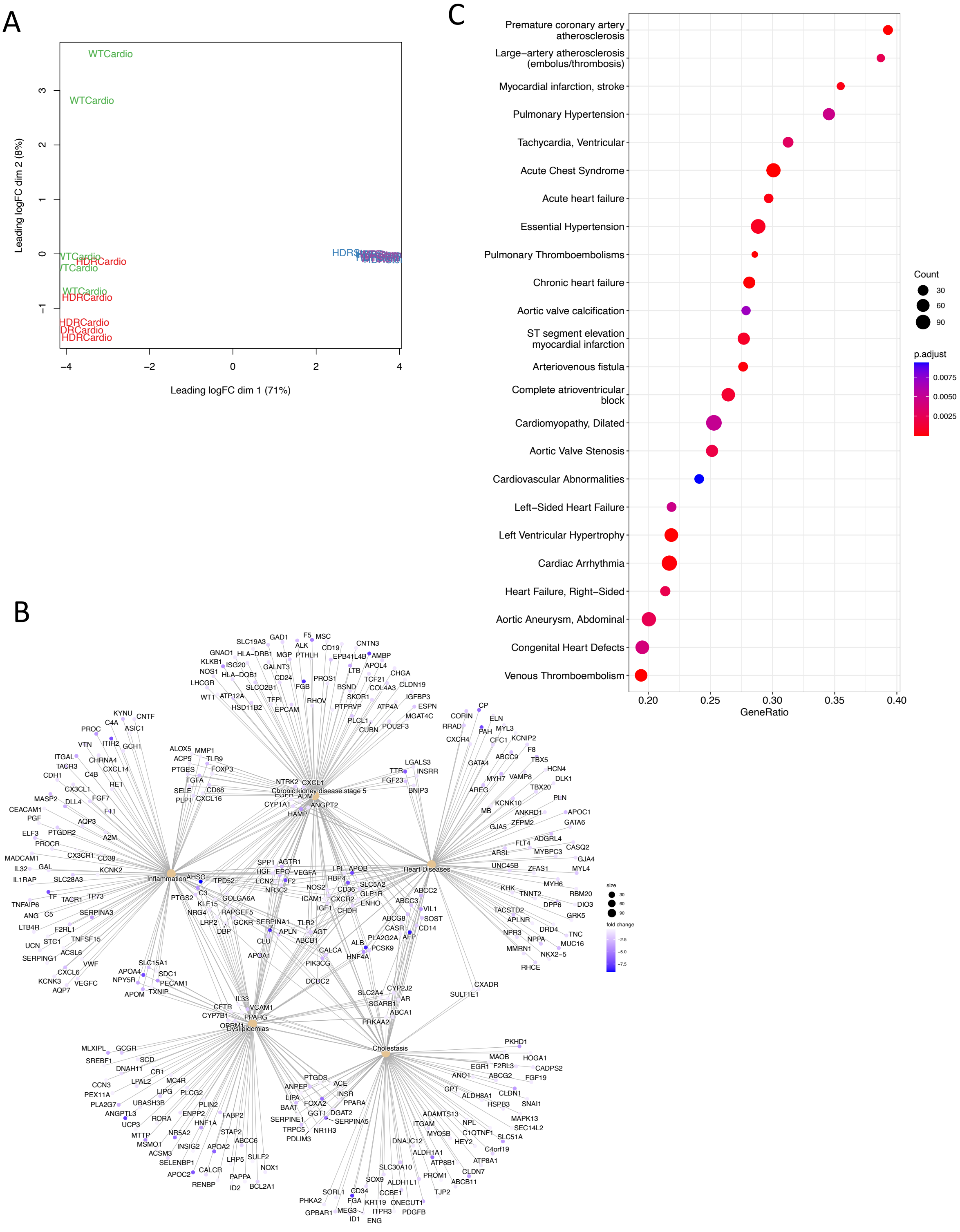

Figure S5. Global changes comparing, and contrasting, *GATA4*_WT and *GATA4*_HDR cells. A, Principal component analysis indicating iPSCs and cardiomyocytes for *GATA4*_WT or *GATA4*_HDR cells. B, Difference in differentiation gene network changes. C, Additional cardiac terms in DisGenNet Enrichment for comparison of cardiac differentiation between *GATA4*_WT or *GATA4*_HDR.

A

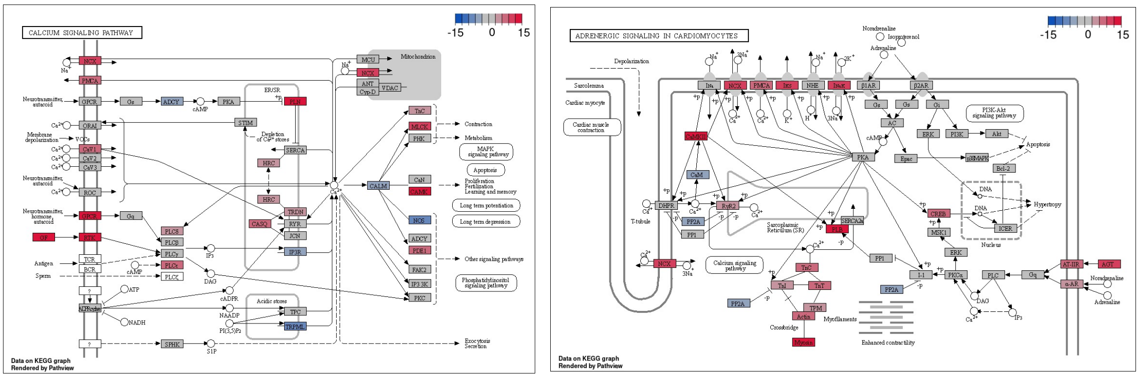

B

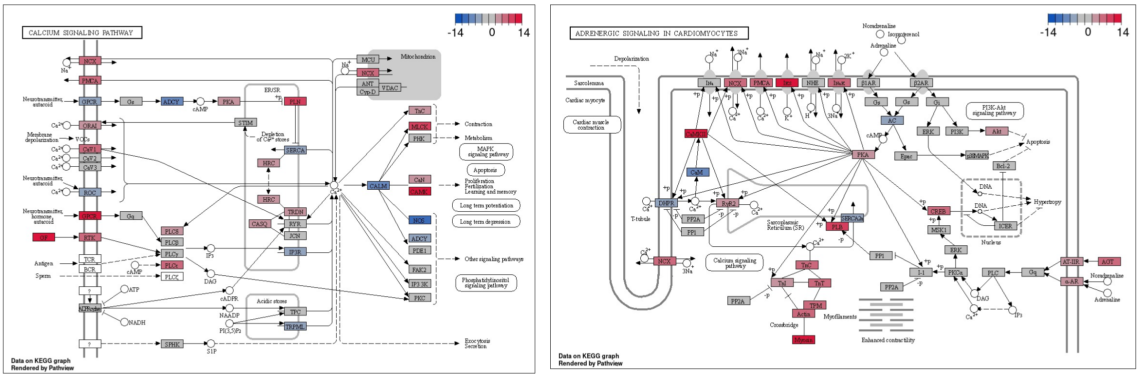

Figure S6. Calcium and adrenergic signaling changes in cardiac differentiation. Gene set enrichment analysis using KEGG pathways. A. *GATA4*_WT iPSC to cardiomyocyte differentiation. B, *GATA4*_HDR iPSC to cardiomyocyte differentiation.

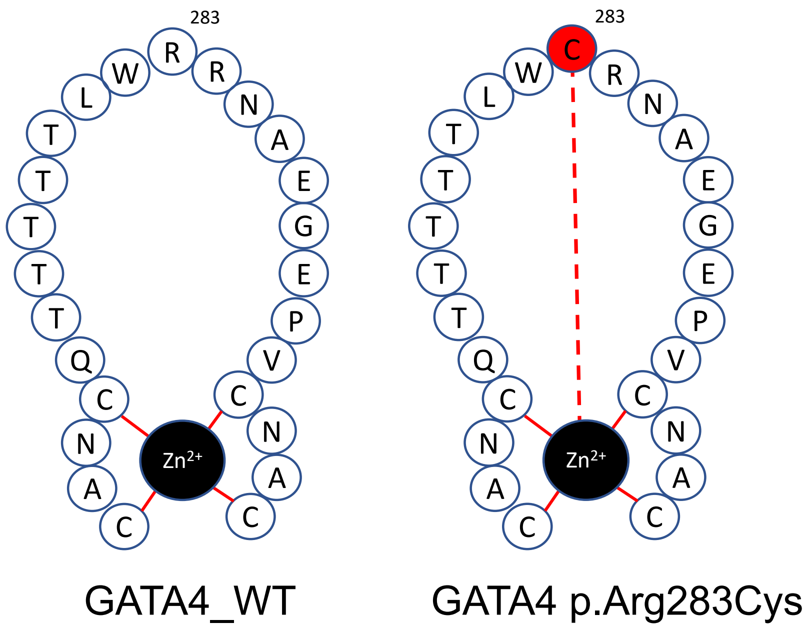

Figure S7. Altered Zn finger domain in GATA4 pArg283Cys protein.

Supplemental Tables.

Differential Gene Expression results for comparisons:

Table S1A. Wild type comparing cardiomyocyes to stem (GeneLevelDE_diff_WT_Cardio_Stem)

Table S1B. HDR comparing cardiomyocyes to stem (GeneLevelDE_diff_HDR_Cardio_Stem)

Table S1C. Difference during differentiation (GeneLevelDE_diff_HDR_WT_Cardio_Stem)

Gene set enrichment results:

Table S1D. GO term significantly enriched in wild type and HDR differentiation (GOTermEnrichedDifferentiation)

Table S1E. DisGenNet terms enriched in differences in differentiation comparison (DisGenNetEnrichedWT_HDR)

Table S1F. Kegg pathways enriched in differences in differentiation comparison (KEGGenrichedDifffWt_HDR)

DataS1G. Geneset DB regulation in differences in differentiation comparison (GenesetDBregDiffWT_HDR)

DataS1H. Geneset Hallmark in differences in differentiation comparison (GenesetHallmark-DiffWT_HDR)
